# Deaminase-Driven Reverse Transcription Mutagenesis in Oncogenesis: Critical Analysis of Transcriptional Strand Asymmetries of Single Base Substitution Signatures

**DOI:** 10.3390/ijms26030989

**Published:** 2025-01-24

**Authors:** Edward J. Steele, Robyn A. Lindley

**Affiliations:** 1 Melville Analytics Pty Ltd. and Immunomics, Kangaroo Point, Brisbane 4169, Australia; 2 Department Clinical Pathology, Victorian Comprehensive Cancer Centre (VCCC), University of Melbourne, Melbourne 3052, Australia; robyn.lindley@unimelb.edu.au

**Keywords:** origin somatic mutations, COSMIC SBS cancer signatures, AID/APOBEC and ADAR deaminases, reverse transcription, immunoglobulin somatic hypermutation, DNA polymerase eta, DNA polymerase theta

## Abstract

This paper provides a critical analysis of the molecular mechanisms presently used to explain transcriptional strand asymmetries of single base substitution (SBS) signatures observed in cancer genomes curated at the Catalogue of Somatic Mutations in Cancer (COSMIC) database (Wellcome Trust Sanger Institute). The analysis is based on a deaminase-driven reverse transcriptase (DRT) mutagenesis model of cancer oncogenesis involving both the cytosine (AID/APOBEC) and adenosine (ADAR) mutagenic deaminases. In this analysis we apply what is known, or can reasonably be inferred, of the immunoglobulin somatic hypermutation (Ig SHM) mechanism to the analysis of the transcriptional stand asymmetries of the COSMIC SBS signatures that are observed in cancer genomes. The underlying assumption is that somatic mutations arising in cancer genomes are driven by dysregulated off-target Ig SHM-like mutagenic processes at non-Ig loci. It is reasoned that most SBS signatures whether of “unknown etiology” or assigned-molecular causation, can be readily understood in terms of the DRT-paradigm. These include the major age-related “clock-like” SBS5 signature observed in all cancer genomes sequenced and many other common subset signatures including SBS1, SBS3, SBS2/13, SBS6, SBS12, SBS16, SBS17a/17b, SBS19, SBS21, as well as signatures clearly arising from exogenous causation. We conclude that the DRT-model provides a plausible molecular framework that augments our current understanding of immunogenetic mechanisms driving oncogenesis. It accommodates both what is known about AID/APOBEC and ADAR somatic mutation strand asymmetries and provides a fully integrated understanding into the molecular origins of common COSMIC SBS signatures. The DRT-paradigm thus provides scientists and clinicians with additional molecular insights into the causal links between deaminase-associated genomic signatures and oncogenic processes.

## 1. Introduction

The purpose here in the Introduction is to provide the conceptual background to the molecular analysis that follows (Section 2), that is, our critical analyses of the underlying causes of the strand biased somatic mutation signatures documented in thousands of cancer genomes.

It is now generally agreed that the cytosine (AID/APOBEC) and adenosine (ADAR) deaminases targeting DNA and RNA C-site and A-site substrates play important roles in health and immunity. However, when dysregulated to “off-target” genomic C- and A-sites in protein coding genes they can potentially cause somatic mutations and add to the severity of progressive genetic diseases such as cancer. Their causative role in front-line innate immunity to viral infections, restriction of dangerously active mobile retrotransposons, as well as mutagenesis of the cancer genome at “off-target” DNA and RNA substrates has been reviewed in detail for AID/APOBEC family of deaminases [1,2]. We have focused over the past 20 years on the putative wider impact of aberrant immunoglobulin (Ig) somatic hypermutation (SHM)-like processes also via ADAR1/ADAR2 deaminase A-to-I editors causing A-to-I(G) mutations in both RNA and DNA substrates across the cancer genome. Thus, a role for both AID/APOBEC and ADAR deaminases in targeted somatic mutations (TSM) in codon context in the TP53 DNA binding region in TP53-negative breast cancers was initially reported [3]. These unconventional TSM analyses also show a putative role in human genomic evolution for AID/APOBEC and ADAR deaminases in the appearance of the many single nucleotide polymorphisms (SNPs) in large numbers of defective genes curated in the human OMIM database (Online Mendelian Inheritance in Man) [4]. All these “off-target” dysregulated AID/APOBEC/ADAR mutagenesis data and others have been comprehensively reviewed [5]. Further, these concepts have inspired promising prognostic/prediction algorithms for a number of cancers [6]. We discuss all these types of cancer mutagenesis data in the context of the reverse transcriptase (RT) mechanism of immunoglobulin (Ig) somatic hypermutation (SHM) at both Ig and non-Ig loci [7].

**Figure 1:**
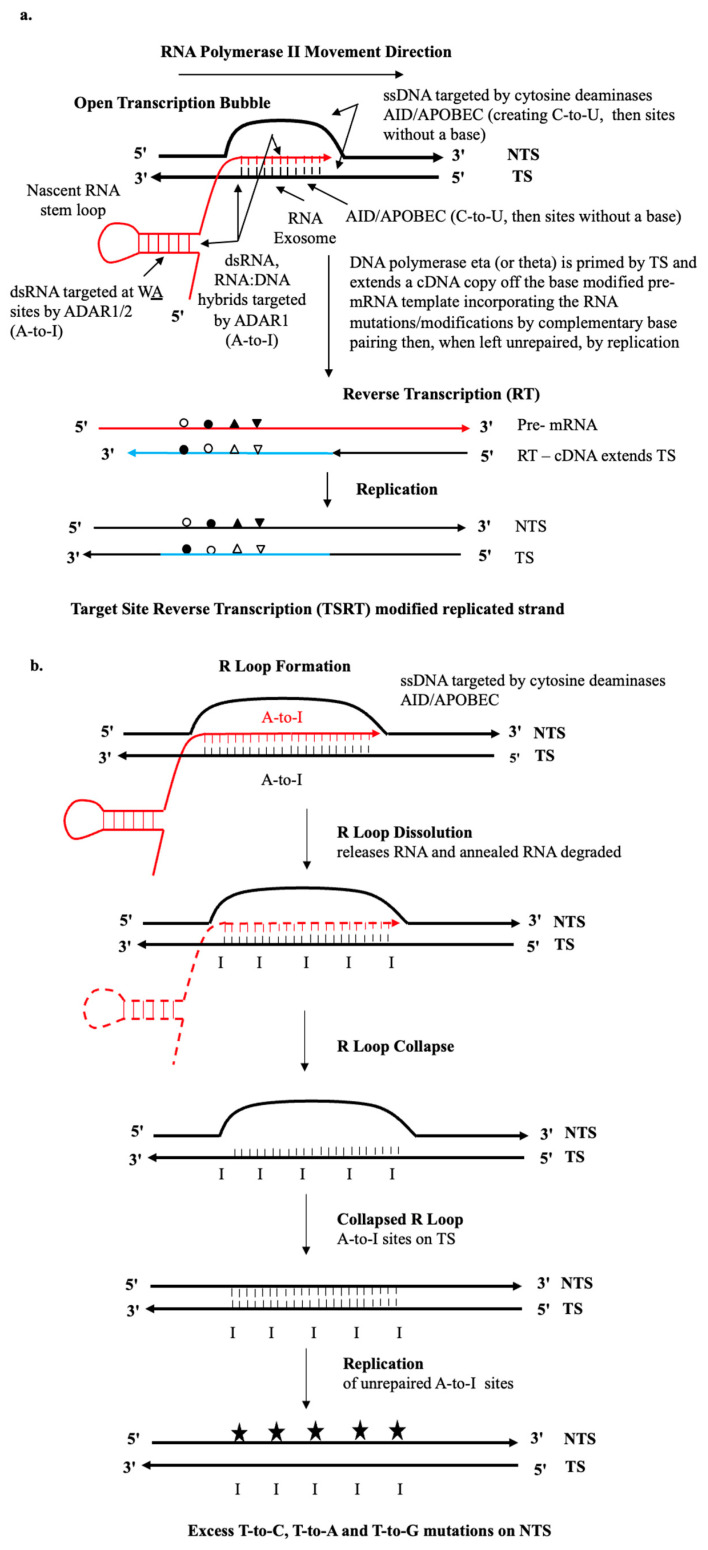
**DRT Model Cancer Mutagenesis** Main substrates and single base substitutions with strand bias consequences for deamination-driven reverse transcription (DRT) mutagenesis in progressing cancer genomes is shown for: (**a**). ***Stalled Transcription Bubble***. The ssDNA sites in the open transcription bubble are targeted by the AID/APOBEC cytosine deaminases and create C-to-U and abasic lesion sites. Black strands represent DNA. Red strands represent RNA. Blue strands represent cDNA. RNA mutations (G-to-A, G-to-C, G-to-U) appear as a consequence of transcription across these AID/APOBEC cytosine deamination lesion sites [15] by the RNA Polymerase II elongation complex (RNA Pol II) on the transcribed strand (TS) indicated by open circles. The RNA exosome allows access to unpaired cytosines on the TS in RNA:DNA hybrid [16]; or by transcription-coupled ADAR1 deamination of adenine to inosine (A-to-I) in the nascent dsRNA or on both nucleic moieties of the annealed RNA:DNA hybrid (9–11 nt) indicated by closed circle. Other subsidiary non-deaminase-driven RNA modifications could include endogenous uracil isomerization to pseudouridine (ψ) to give a U-to-G miscoding substitution [17,18,19,20], indicated as closed triangles; or non-deaminase-driven RNA miscoding mutations (G-to-U) following reactive oxygen species (ROS) generation of 8oxoG (c.f. SBS18 transcriptional strand asymmetry) in nascent RNA or the annealed RNA:DNA hybrids [21], indicated by inverted closed triangles. The last TSRT step is effectively a potential “error prone” DNA repair process akin to a patch nucleotide excision repair (NER) on the TS allowing replication of the helix in that damaged genomic region, discussed at length in figure four in Franklin et al. [22]. Alternate symbol fills are shown to symbolize RNA mutation or modification as a complementary base pairing partner in DNA. Also see and compare the prior published schematic summary showing the main elements of the reverse transcriptase (RT) mechanism for immunoglobulin (Ig) somatic hypermutation (SHM)—RT Ig-SHM—and the target site reverse transcription (TSRT) process as a patch correction around DNA lesion sites following Luan et al., 1993 [23] as discussed by Steele et al., 2024 [7] and Figure 2. The term “ Then sites without a base” is also called an abasic site. (**b**). ***R Loops***. See text for more detail on deamination modifications by ADAR1 or ADAR2 [24,25] at long (40 nt–670 nt) annealed RNA:DNA hybrids at R Loops [26]. Black strands represent DNA. Red strands represent RNA. These are often generated under replicative stress in the body of the genome, particularly at transcription replication fork collisions or conflicts (TRCs) on the same strand [25,26] at deaminated A-sites in both the RNA and DNA moieties. These DNA A-to-I modifications are also referred to as hypoxanthine (Hx). As discussed in the text, such deaminations contribute to R Loop dissolution by facilitating the release of the firmly bound RNA and then its degradation by RNaseH activity. After R Loop collapse, the inosine modified TS (Hx) sites remaining unrepaired will be replicated over and result in excess T-to-C, T-to-A and T-to-G mutations (filled stars) on the NTS. The incidence of these mutations (in order T-to-C>T-to-A>T-to-G) result in transcriptional strand asymmetry signatures as discussed in detail in the text and summarized in Table 3.

This paper, therefore, lays out a critical analysis of the mechanisms of origin of transcriptional strand asymmetries observed in the single base substitution (SBS) signatures curated at the online COSMIC database (Box 1, Figure 1, Table 1). One subsidiary aim is that this knowledge may also be leveraged to develop more precise predictive genomic tests for use in the clinic and for understanding personalized medicine in patient responses to different cancer treatments, such as in recent machine learning analysis of available genomic sequence data on many cancers [6], as well as the prior work on prognostic/predictive codon context-based targeted somatic mutations (TSM) in High Grade progressing ovarian cancers, HGS-OvCa [8].

**Table 1:** Somatic point mutation patterns (as a percentage of the total) in data sets involving rearranged murine *IgV* loci (**A**) and in human cancer SBS5 (**B**), SBS3 (**C**).

**A. Somatic mutations (Mean % 12 Studies Plus SEM) in Rearranged Murine *IgV* Loci**						
		**Mutant Base**				
**From**	**A**	**T**	**C**	**G**	**Total**	**Strand Bias Factor**
**A**		10.6 (1.2)	6.3 (0.9)	14.6 (0.7)	31.6 (1.7)	A>>T 2.9×
**T**	3.1 (0.6)		5.3 (1.1)	2.6 (0.6)	11.0 (1.3)	*p* < 0.001
**C**	4.3 (0.8)	13.4 (1.3)		3.6 (0.7)	21.3 (1.3)	G>>C 1.7×
**G**	20.1 (1.9)	7.2 (1.4)	8.7 (0.7)		36.1 (2.5)	*p* < 0.001
**B. Somatic Mutations (as Percentage of Total 89,120 Mutations) in SBS5**						
		**Mutant Base**				
**From**	**A**	**T**	**C**	**G**	**Total**	**Strand Bias Factor**
**A**		5.3	3.7	16	25	A>>T 1.1×
**T**	4.9		13.9	4.3	23.1	*p* < 0.001
**C**	5.4	15.5		4.2	25.2	G>>C 1.1×
**G**	15.9	6.5	4.2		26.5	*p* < 0.001
**C. Somatic Mutations (as Percentage of Total 53,833 Mutations) in SBS3**						
		**Mutant Base**				
**From**	**A**	**T**	**C**	**G**	**Total**	**Strand Bias Factor**
**A**		8.4	4.7	8.8	21.8	A>>T 1.04×
**T**	7.8		8.1	5.2	21	*p* > 0.05
**C**	9.3	8.4		9.5	27.2	G>>C 1.1×
**G**	9.3	10.9	9.8		29.9	*p* < 0.001

All data rounded to one decimal place. All mutations are read from the coding or non-transcribed strand (NTS). A. Data from Steele 2009 [9]. B. and C. Data from Alexandrov et al., 2013, 2020 [10,11]—Single Base Substitution Mutational Signatures (v3.4 October 2023) at the COSMIC website at https://cancer.sanger.ac.uk/signatures/sbs/sbs5/ (accessed on 15 April 2024) and https://cancer.sanger.ac.uk/signatures/sbs/sbs3/ (accessed on 15 April 2024). Only cancer types with a minimum 2000 mutations for the SBS5 or SBS3 signatures with average probability at least 0.75 are considered, for real mutations on transcribed and non-transcribed strands. In both B, C, a Chi-square 4 × 4 test (assigning a nominal 10 to empty cells) gives very large Chi-square values with *p*-values <0.00001. In B, mutations of T-to-G significantly exceed mutations of A-to-C by 1.16× *p* < 0.001. In C, there are strand biases within A:T base pairs where A-to-G mutations exceeds T-to-C mutations by 1.1× giving *p* < 0.01. Similar data for SBS5 and SBS3 broken down by cancer tissue type are shown in Tables S1 and S2. Generic symbol A>>T means mutations of A exceeding mutations of T at A:T base pairs. Generic symbol G>>C means mutations of G exceeding mutations of C at G:C base pairs.

Box 1 **COSMIC SBS Signatures—Main Summary by C-site, A-site Category.**

Alexandrov and colleagues [10,11,12] report single base substitution signatures (SBS) of mutagenesis in cancer using an algorithm-extraction of tri-nucleotide signatures from whole exome (WES) and whole genome (WGS) sequence data from thousands of cancer genomes. The present categories offer an alternative way of understanding these somatic mutation patterns based on the likelihood of the origin of the dominant deaminase-driven signature—C-site or A-site, or both C-site plus A-site. This categorisation is cognisant of the fact that certain apparent non-deaminase driven or ‘environmental exposure’ signatures (Tobacco Smoking, UV exposure, Reactive Oxygen Species viz. 8oxoG) are also present in certain cancer genomes. The most dominant signature is SBS5 occurring in all cancer genomes sequenced. SBS5 displays a ‘dysregulated Ig-Like SHM’ somatic mutation pattern [1,7,8,9,13,14] with transcriptional strand bias of mutations of A exceeding mutations of T (A>>T) and mutations of G exceeding mutations of C (G>>C), as in Table 1, Table 2 and Table 3, online Supplementary Tables S1–S3. This strand-biased mutation pattern is also observed at both Ig and non-Ig loci (the TP53 DNA binding region) that have undergone somatic mutagenesis [7,14]. Listed below are the authors’ main Deaminase Driven Categories. Superscript T indicates Transcriptional Strand Asymmetry. The SBS Mutational Signatures (v3.4—October 2023) are at The Catalogue of Somatic Mutations in Cancer (COSMIC) website at https://cancer.sanger.ac.uk/signatures/sbs/ (accessed on 15 April 2024)**C-site predominantly (putative AID/APOBEC driven)****SBS1^T^** (at ACG strong strand bias, 5-meCpG, but slight reverse C>>G at CCG, GCG, TCG), **SBS2, SBS6, SBS13, SBS19^T^** (pure almost G>A >>C>T only), **SBS7a, SBS7b****A-site predominantly (putative ADAR1 and coupled Target Site Reverse Transcription [TSRT], Pol-Eta and/or possibly DNA Pol-Theta driven)****SBS12^T^**(Liver), **SBS16^T^** (Liver), **SBS17a^T^**, **SBS17b^T^** (but reverse strand bias not A>>T it is T>>A), **SBS21^T^** (DNA mismatch repair deficiency, but apparent reverse strand bias not A>>T it is T>>A)**C-site plus A-site more or less balanced “Ig-SHM-like” (AID/APOBEC/ADAR driven + TSRT via Pol eta, Pol theta?) SBS5^T^ (SBS40 = SBS5?), SBS3^T^, SBS9^T^** (but apparent reverse strand bias not A>>T it is T>>A).SBS signatures not highlighted in above categories are discussed and analysed at length in Results and Analytical Discussion Section 2.1

**Table 2:** SBS5: Strand biases in types of mutations in different cancers.

			Strand Bias at Selected Base Pairs			
	Global Strand Bias		A-to-G>	T-to-G>	G-to-A>	G-to-T>
Cancer	A>>T	G>>C	T-to-C	A-to-C	C-to-T	C-to-A
Billiary-AdenoCA	+++	+++	+++	++	+	++
Bladder-TCC	+++	+++	+++	++	+++	+++
Breast-Cancer	+++	+++	+++	+++	+++	+++
CNS-GBM	++	+++	+++	+	+++	+++
CNS-Medullo	+	+++	+++	++	+++	+++
ColoRect-AdenoCA	RNS	+++	R+	+++	+++	+++
ESCC	+++	+++	+++	+++	+++	+++
Eso-AdenoCA	NS	+++	NS	++	NS	+++
Head-SCC	+++	+++	+++	NS	+++	+++
Liver-HCC	+++	+++	+++	+++	+++	+++
Lung-AdenoCA	+++	+++	+++	+++	NS	+++
Lung-SCC	+++	+++	+++	R++	+++	+++
Lymph-BNHL	NS	+++	NS	+	+++	+++
Lymph-CLL	+++	+	+++	+++	++	+
Panc-AdenoCA	+++	+++	+++	+	+	+++
Prost-AdenoCA	+++	+++	+++	+++	NS	+++
Skin-Melanoma	R++	+++	+++	+++	++	+++
Stomach-AdenoCA	NS	+++	+++	+++	+	+++
Uterus-AdenoCA	R+++	++	+	+++	NS	+

Code: Intensity Metric of Strand Bias +++ means *p* < 0.001, ++ *p* < 0.01, + *p* < 0.05, NS *p* > 0.05. R is Reverse Direction of Dominant Global Strand Bias. These summaries have been constructed from the data in Table S1. Only those cancers where the total number of mutations is approx. 30,000 are shown. The flips and inconsistency of direction C-to-G>G-to-C and C-to-G<G-to-C consistent with REV1 translesion DNA repair equally focused on repairing single bp lesions on both strands [9]. To summarize C-to-G>G-to-C is NS Billary-AdenoCA, Bladder-TCC, CNS-GBM, ColoRect-AdenoCA, Es0-AdenoCA, Lymph-BNHL, Skin-Melanoma, Stomach-AdenoCA, Uterus-AdenoCA, and C-to-G>G-to-C is significant at least *p* < 0.05 in Breast-Cancer, Liver-HCC, Lymph-CLL, Panc-AdenoCA, Prost-AdenoCA, and C-to-G<G-to-C at least *p* < 0.05CNS-Medullo, ESCC, Head-SCC, Lung-AdenoCA and Lung-SCC.

**Table 3:** Summary of origins and features of main SBS types.

			Transcriptional Strand Asymmetry	
	Deduced Deamination		Inferred Cause of Transcriptional Strand Asymmetry	Inferred Cause of T-to-C>A-to-G at Collapsed R Loops †
	DNA	RNA		
COSMIC SBS	C-to-U	A-to-I		
SBS5	AID/APOBEC	ADAR (+Hx)	TSRT	ADAR (Hx)
SBS1	AID/APOBEC		TSRT	
SBS2/SBS13	AID/APOBEC			
SBS3	AID/APOBEC	ADAR	TSRT, TCR	
SBS4			TCR	
SBS6	AID/APOBEC			
SBS7a, SBS7b	AID/APOBEC		TCR	
SBS7c, SBS7d		ADAR (+Hx)		ADAR (Hx)
SBS8			TCR	
SBS9	AID/APOBEC	ADAR (+Hx)	TSRT	ADAR (Hx)
SBS10a,b SBS14	AID/APOBEC			
SBS11	AID/APOBEC		TSRT	
SBS12	AID/APOBEC	ADAR	TSRT	
SBS15	AID/APOBEC			
SBS16		ADAR	TSRT	
SBS17a, SBS17b		ADAR (+Hx)		ADAR (Hx)
SBS18			TSRT	
SBS19	AID/APOBEC		TSRT	
SBS84	AID/APOBEC			
SBS85		ADAR (+Hx)		ADAR (Hx)

† In some cases, depending on context, Wobble Base pairing by Hypoxanthine (Hx) or A-to-I deaminated adenine on the template or transcribed DNA strand (TS) may also lead to excesses of T-to-A>A-to-T, or even T-to-G>A-to-C post replication on the non-transcribed strand (NTS) as Figure 1 e.g., SBS9, SBS7c,7d. For further information on the origin of each signature see text Section 2. It should be noted that there are many overlapping target motifs for AID/APOBEC and ADAR deaminations that reflect uncertainty in the field on the exact hierarchy of the targeting preferences, suggesting a “deaminase overlay” in many somatic mutation cancer signatures (which is implied by the analysis of the origins of global signature SBS5). This has been reviewed [5], and the main tabular deaminase motif target summary is in Table S4. TSRT, target site reverse transcription as Figure 1a; TCR, conventional transcription coupled repair; APOBEC, apolipoprotein B mRNA-editing, catalytic polypeptide; AID, activation-induced cytidine deaminase, a member of APOBEC family of cytosine deaminases; ADAR, adenosine deaminase acting on RNA.

### 1.1. AID/APOBEC and ADAR Ig SHM-like Dysregulated Mutagenesis

We hypothesize that transcriptional strand asymmetries in SBS signatures can be understood by the action of the mutagenic cytosine (AID/APOBEC) and adenosine (ADAR1/2) deaminases (see Figure 1) often coupled to cellular reverse transcription allowing the generation of distinct transcriptional mutation strand biases. In the case of A-to-I pre-mRNA editing via ADAR1 [27], there is an implied association with cellular reverse transcription, via DNA repair Polymerases eta and theta [7,28] (see Figure 1a). DNA replication of ADAR deaminase-mediated A-to-I DNA modifications (A-to-Hx, Hypoxanthine) can also help explain, as the present analysis shows, distinct strand-biased outcomes at resolved (collapsed) long transcriptional R-Loops. An R-loop, in contrast to an RNA Pol II driven Transcription Bubble is a very long three-stranded nucleic acid structure, composed of a long annealed DNA:RNA hybrid and the associated displaced non-template single-stranded DNA (see Figure 1b).

Additionally to the main SBS list in Box 1, there are some secondary downstream mutation signatures [10,11,12], such as Defective Homologous Recombination Repair (dHR; SBS3), defective DNA mismatch repair (dMMR; SBS15, SBS21, SBS26, SBS44), defective base excision repair (dBER; SBS30, SBS36), defective nucleotide excision repair (dNER), and defects in replicative polymerases *POLE* or *POLD1* genes (dPOLE/dPOLD1; SBS10a,10b, SBS14, SBS20) that may result in additional replication fork-based strand-biased signatures (which is not our focus). However, in the majority of these cases it is posited here that the primary source of the de novo somatic mutations is associated with deaminase mutagenic activity: either a C-to-U, C-to-T, or A-to-I modification potentially causing a mutagenic outcome in DNA or RNA sequence of the cancer genome or transcriptome (which can subsequently, if left unrepaired, be copied back to the evolving cancer genome via cellular reverse transcription). This interpretation assigns causative “AID/APOBEC activity” to a far wider set of SBS signatures than is currently allocated at the online COSMIC site [11,12] to just SBS2 and SBS13.

In this paper the deaminase-driven reverse transcriptase (DRT) paradigm is formerly introduced to show how the above scenarios can plausibly occur in a transcription-linked path during oncogenesis (See Supplementary File Sections S1 and S2 for a wider historical background to the current analysis including a longer list of abbreviations and definitions). It provides a molecular analytical framework based on molecular biology first principles of DNA replication, RNA transcription, and DNA repair. It is a set of foundation features and assumptions that involve AID/APOBEC and ADAR deamination coupled in many cases to a target site reverse transcription, TSRT [23]. It includes the RT activity of the DNA repair polymerase-eta, with putative back up across the cancer genome by the RT activity of DNA repair polymerase-theta [7,28]. This is a clear variation in known RNA templated DNA repair processes now documented in yeast (*Saccharomyces cerevisiae*) and in human embryonic kidney cells lines (HEK293 cells)—see Section 1.5. Therefore, while this significant step is still not fully understood in every molecular detail, the TSRT process at DNA mutational lesions allows RNA A-to-I mutational modifications to be fixed back into the genomic DNA, scoring primarily as an A-to-G mutation at that site when this unrepaired I (Inosine) is accurately copied and replicated. For example, it helps our understanding of the genesis of the striking transcriptional strand biased A-to-G mutations observed in the genomic DNA of liver cancer cells at WA sites (e.g., origins SBS12, Section 2.1.12).

Other prominent endogenous mutation sources of note include reactive oxygen species (ROS) elevated in Innate Immune Responses and the cell-wide stress response initiated by Interferon-Stimulated Gene cascades, which can also activate APOBEC and ADAR deaminases [29]. ROS can result in oxidative 8oxoG modifications that lead to primary G-to-T mutations (SBS18) that are particularly prominent in Brain and CNS abnormalities [30] and some other cancers [11]. Other important endogenous alkylating events at G, A, T bases may result in non-bulky base modifications that cause instructive mutagenic lesions, including 06-meG G-to-A (C-to-T), 04-meT T-to-C (A-to-G) or cytotoxic lesions (N-7-meG, N3-meA, N2-meG). These often result in abasic sites and ssDNA nicks that are expected to be repaired by a base excision repair (BER) step [31,32].

The deaminase-driven reverse transcriptase (DRT) hypothesis was first articulated in part in 2010 [13]. It then developed further when applied to understanding the transcriptional strand biases of C-to-U(T) mutations at G:C base pairs and accompanying targeted mutations occurring at A:T base pairs in the DNA binding region of TP53-ve tumor samples [3,14]. The principles of the DRT hypothesis, as now formally articulated here for cancer mutagenesis, were further employed in toto or in part in subsequent prediction/prognostication analyses by applying more specific codon-context targeted somatic mutation (TSM) analysis to tumor-normal NGS tumor-normal NGS sequence data [8], and in other deaminase-based somatic mutation and genetic analyses [4,6,33].

The main difference between the DRT hypothesis and other diagnostic and therapy-focused deaminase-associated signature analyses [10,11,12,34] is that the DRT-paradigm focuses on the two main types of mutator processes in carcinogenesis (Box 1). These are: (a) Mutagenic C-site deaminations AID/APOBEC (C-to-U, and C-to-T at 5′meCpG sites); (b) A-site deaminations mediated by ADAR1/2 RNA A-to-I editors (read as A-to-G). In most other oncogenic signature analyses involving transcriptional strand asymmetry, the latter is often ignored or overlooked. This may have been because in the past, the reverse transcriptase model of Ig SHM itself has been controversial, yet that controversy has now died down as more independent data have accumulated [7,28] together with the recognition that the general phenomenon of RNA templated DNA repair, which is now accepted by the non-immunological biochemistry research community working on RNA directed DNA repair mechanisms (see Section 1.5).

**Figure 2:**
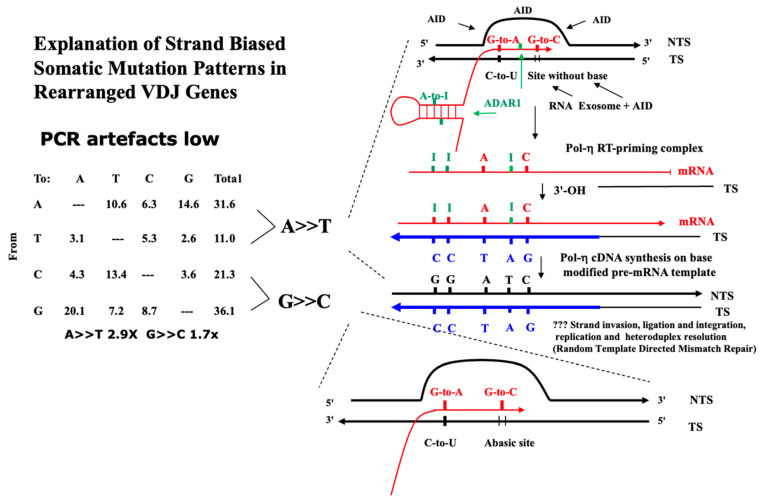
**The reverse transcriptase mechanism of strand-biased immunoglobulin somatic hypermutation (RT-Ig SHM)**. A schematic outline showing the main mutational events at Stalled Transcription Bubbles generated by RNA Pol II [7]. Black strands represent DNA. Red strands represent RNA. Blue strands represent cDNA. The green sites are A-to-I RNA editing sites in double stranded RNA or in both RNA and DNA moieties of RNA:DNA hybrids. Site without a base is also called an abasic site.

The detection of assumed RNA deaminations at ADAR-targeted WA sites now apparent in genomic DNA thus results from the coupling to cellular reverse transcription (DNA Polymerase eta and now putatively DNA Polymerase theta) at many non-Ig loci across the cancer genome. It, therefore, follows that the execution of TSRT with the integration of an error-filled cDNA copy of the base modified transcribed strand (TS) provides the most plausible explanation for understanding how oncogenic strand bias mutation patterns involving both C-site and A-site base modifications arise. This extends 5′ and 3′ as a variable length integrated cDNA “patch” around the deaminase lesion site in the genomic DNA as summarized (Figure 1a) and as developed from the reverse transcriptase mechanism of Ig-SHM [7] (Figure 2). However, it needs to be made clear at this juncture that ADAR-mediated A-to-I deamination can also occur in principle at WA sites (AA or TA) directly on the DNA moiety of annealed RNA: DNA hybrids [35] that are ubiquitously generated at Transcription Bubbles and R-Loops (Section 1.3 and Section 1.4).

Before proceeding to the detailed analysis of the likely origins of the major SBS signatures (Section 2), the main nucleic acid substrates for AID/APOBEC and ADAR deamination first need to be discussed.

### 1.2. Lagging and Leading Strands of the Replication Forks

These are a significant source of unpaired and exposed ssDNA for AID/APOBEC mediated mutations at C-sites in various SBS signatures. However, they are not strictly relevant to understanding “Transcriptional strand asymmetries” and are not directly discussed or analyzed in detail here, but they are discussed in more detail in Supplementary file Section S2A.

### 1.3. Stalled Transcription Bubbles in RNA Pol II Transcribed Regions

These provide the great bulk of “Transcriptional strand asymmetries” observed in SBS signatures and are a genome-wide rich source of DNA and RNA substrates for somatic mutations [5,6,36]. Open Transcription Bubbles provide ssDNA in the displaced non transcribed strand (NTS), as shown in Figure 1a and Figure 2. This allows access to C-to-U DNA deamination in the context of the key variable deaminase motifs often close by and overlap, particularly in Ig variable regions: AID at WRCN motifs; various APOBEC3 family members at TCN motifs (APOBEC3A, APOBEC3B, APOBEC3H); and, CCN motifs (APOBEC3G). On the template transcribed strand (TS), in addition to ssDNA tracts at the 5′ and 4′ edges of the bubble, the RNA Exosome actively permits access to unpaired C-sites in the annealed RNA:DNA hybrids [16]. Stalled Transcription Bubbles would also allow the annealed RNA:DNA hybrid region to be attacked by ADAR1 or ADAR2 acting on adenosines base paired in both dsRNA or DNA and RNA moieties of the DNA:RNA hybrid [35]. The nascent dsRNA in stem-loops emergent from the Transcription Bubble also present deamination targets for the transcription coupled Z-DNA binding by ADAR1 associated with RNA Pol II elongation [27]. APOBEC3A is also a known RNA C-to-U editor [1,37,38] and can in theory deaminate nascent pre-mRNA molecules. Stalled Transcription Bubbles are widespread and high frequency events in all protein coding RNA Pol II transcribed genes studied—from the transcription start site (TSS) to a point about 3Kb downstream into the genic regions [39].

### 1.4. Long R-Loops in RNA Pol II Transcribed Regions

R-Loops, in contrast to shorter Stalled Transcription Bubbles, offer a major source of both long unpaired ssDNA and long annealed RNA:DNA hybrid substrates [5,6,26,35] for both AID/APOBEC C-to-U and ADAR1/2 A-to-I deaminations. ADAR2 has been shown in vitro to deaminate both RNA and DNA moieties of the RNA:DNA hybrid [35]; and the ongoing work by Tasakis et al., 2020 (Pers comm N.F. Papavasiliou) reveals direct ADAR DNA deaminations at RNA:DNA hybrids within R-Loops in vivo (Figure 1b), in progressing multiple myeloma [40]. It has been reported that APOBEC3B both regulates R-Loop formation and promotes transcription-associated mutagenesis in cancer [41]. The entire APOBEC3 family is under TP53 expression regulatory control [42], and we also expect that the RNA editing properties of APOBEC3A play a similar role in RNA:DNA hybrid collapse and resolution (Figure 1b) as it is also a major C-to-U DNA editor in cancer genomes.

Recent evidence analyzed herein implies that both nuclear ADAR1 and ADAR2 act to resolve long annealed RNA:DNA hybrids by A-to-I editing the DNA moiety *and* RNA moiety (Figure 1b). This facilitates the release of the annealed nascent RNA moiety, which then becomes susceptible to digestion by RNase H enzymes that act by cleaving the RNA released in RNA/DNA hybrids. It is conceivable that APOBEC3A also plays a role in C-to-U editing of the nascent RNA at R-Loops in their dissolution. Such RNA and DNA modifications are expected to assist the collapse of R-Loops to dsDNA helices, *albeit* now potentially modified by putative A-to-I DNA modifications in some cases (Hypoxanthine, Hx). If these are left unrepaired followed by replication across the R-Loop collapsed region, it allows T-to-C and other Wobble Pair transversions T-to-A, T-to-G to be fixed on the NTS of the DNA helix (Figure 1b).

Three important areas supplying data from unrelated biomedical research over the past 10–20 years now require highlighting before proceeding to the detailed analysis of SBS mutational strand asymmetries. These are (a) the phenomena of RNA templated DNA repair (Section 1.5): (b) experiments showing how AID deaminases actually latch onto and travel with nascent pre-mRNA polymers in RNA Pol II elongation (the “RNA Tether Model”), Section 1.6; and, (c) the very real yet not widely known or understood phenomenon of aberrant ‘Non-B Lymphocyte Ig synthesis and secretion’ in all cancer cells examined thus far, Section 1.7.

### 1.5. Biochemistry of RNA Templated DNA Repair

We highlight the molecular and biochemical data that strongly supports the TSRT mechanism at the core of the DNA Polymerase eta-driven reverse transcriptase mechanism of antigen-driven immunoglobulin somatic hypermutation (RT Ig-SHM, Figure 2) mechanism in Germinal Centre B lymphocytes [7].

These data have emerged over the past 10–15 years from the non-immunological biochemistry DNA repair discipline. The first from the group of Francesca Storici dating to 2007 [43,44,45] on the mechanism of RNA Templated Homologous Recombination (HR) DNA strand break (double or single strand) repair in yeast strains. The responsible cellular reverse transcriptase now identified in the yeast phenomenon is DNA polymerase zeta, which recent data demonstrate is a far more efficient cellular reverse transcriptase in yeast than its DNA repair translesion counterpart DNA polymerase eta [46].

The other definitive and directly relevant work is by the group of Tapas K Hazra [47,48,49]. Their work is in a well-defined DNA repair system with stable human embryonic kidney cell lines (HEK293 cells). These data, particularly in Chakraborty et al., 2023 [49], provide the *most definitive* demonstration showing that transcription-coupled (TC) RNA templated non-homologous end joining repair (TC-NHEJ) of double strand breaks (DSB) is clearly executed in a target site reverse transcriptase process (TSRT), as hypothesized by Andrew Franklin during his PhD in 2003 who first demonstrated reverse transcriptase activity human DNA polymerase eta and other Y family members DNA polymerases iota and kappa [50]. In addition, a quite separate analysis by us on the putative reverse transcriptase origin, via an hypothesized transcription-coupled nucleotide excision repair (TC-NER) of CAGn and related repeat expansions in neurological diseases was published in 2020 by Franklin et al. [22]; its relevance to the present review lies in the fortuitous fact of its timely relation to the work of the Hazra group published independently in the same year on similar in-frame trinucleotide expansion diseases such as spinocerebellar ataxia type-3 [48]. 

### 1.6. The RNA Tether Model

The “RNA Tether Model” of Michael R Lieber and colleagues fundamentally enhances our understanding of the mechanism of “off-target RT Ig-SHM like” processes consistent with our general model of dysregulated AID/APOBEC and ADAR driven RT Ig-SHM like mutagenesis across the cancer genome.

The earlier work on the mechanisms of human chromosomal translocations in fragile zones [51] has now led to the clear and definitive RNA tether model [52]. This model of AID deaminase action is applicable to not only chromosomal translocations, but also potentially generalizable to Ig SHM and Ig Class Switch Recombination (CSR), and thus implied now also to APOBEC1 and APOBEC3 family deaminations in cancer mutagenesis [3,5]. AID proteins can be tethered to *any* nascent emergent RNA during RNA Pol II elongation, leaving their deamination binding domains free to deaminate unpaired cytosines in ssDNA regions in and around Stalled Transcription Bubbles (cf. ssDNA regions as shown in Figure 1a and Figure 2)—or potentially even for nascent RNA targeted C-to-U editing by APOBEC3A. The implication is that provided AID deaminase proteins are in the *same* immediate nuclear vicinity, *any* emergent non-Ig nascent RNA can be subject to “off-target” mutagenesis. The immediate vicinity could mean the coincidence of the non-Ig transcribed gene transcription factory with an “Ig transcription factory”, as discussed in PR Cook and associates (as reviewed recently in Steele [53]). Therefore, the RNA tether model is a major conceptual advance. It is directly relevant to understanding “dysregulated Ig SHM-like AID/APOBEC/ADAR mutagenesis” across the cancer genome as discussed here and demonstrated in “hot” and “cold” AID deamination topographical associated domains zones by David G Schatz and colleagues in the Ramos Burkitt Lymphoma derived cells lines [54].

### 1.7. Non-B Lymphocyte Immunoglobulin

The pan-cancer phenomenon of non-B Lymphocyte immunoglobulin (Ig V[D]J DNA Rearrangements) and immunoglobulin synthesis in de novo emergent cancer cells of diverse epithelial origins is also consistent with a model of “dysregulated AID/APOBEC and ADAR driven RT Ig-SHM-like mutagenesis” across the pan-cancer genome.

This is the important work of the group of Xiaoyan Qui and colleagues since 1996. They have characterized the clear reality of cancer-derived Ig proteins emerging de novo in all cancer types examined thus far [55,56,57,58]. Enough independent evidence marshaled by the Qiu group and others convinces the present authors this is a real “Non-B cell Ig” phenomenon of cancer cells (and some transient occasional positivity of some normal cells in the lung and colon). Thus, cancer cell carcinomas derived from the epithelium of the lung, colon, breast, and other tissue sites express and secrete classic Ig molecules, in particular IgG as intact HL heterodimers of 150 Kd of unknown antigen binding specificity. The expressed and secreted Ig is a cancer biomarker [57] and the expressed Ig is associated with pro-tumorgenic properties, metastasis, and tumor evasion. The molecular mechanisms of how all cancers display this phenomenon is poorly understood.

The phenomenon cannot be explained by contaminating infiltrating B lymphocytes or hemopoietic lineage lymphocytic cells—the Ig is clearly cancer cell derived, with genuine heavy chain V[D]J and light VJ rearrangements with overtly functional VDJ and VJ in-frame joints (plus or minus N region additions at the junctions). The patient cancer cells display restricted sets of germline VH rearrangements in sets of VDJ functional rearrangements—and the expressed Ig protein displays aberrant glycosylation in H chain constant regions [58] the assumed glycosylation marker characterized exhaustively by Gregory Lee [57].

An important paper in the series is Zheng et al., 2009 [58], which shows an apparent classic strand bias yet clearly “dysregulated Ig-SHM” occurring de novo in cancer cells. Thus, there are clear somatic (hyper)mutations of these functional rearrangements displaying the signature of Ig SHM strand biased patterns with mutations of A exceeding mutations of T, A>>T (at WA/TW sites, implying a role for DNA Polymerase eta) and mutations of G exceeding mutations of C, G>>C (RGYW/WRCY), as we have reviewed here (Table 1) and elsewhere [7]. This, of course, implies AID-driven reverse transcription mutagenesis in oncogenesis, as discussed in earlier Lindley and Steele papers since 2010 [1,8,13,14].

All this necessary conceptual background now sets the stage for the systematic analysis of the causative origins of the main transcriptional strand biased SBS signatures.

## 2. Results of Critical Analysis of Transcriptional Strand-Bias: Implications for SBS Origins

**Figure 3:**
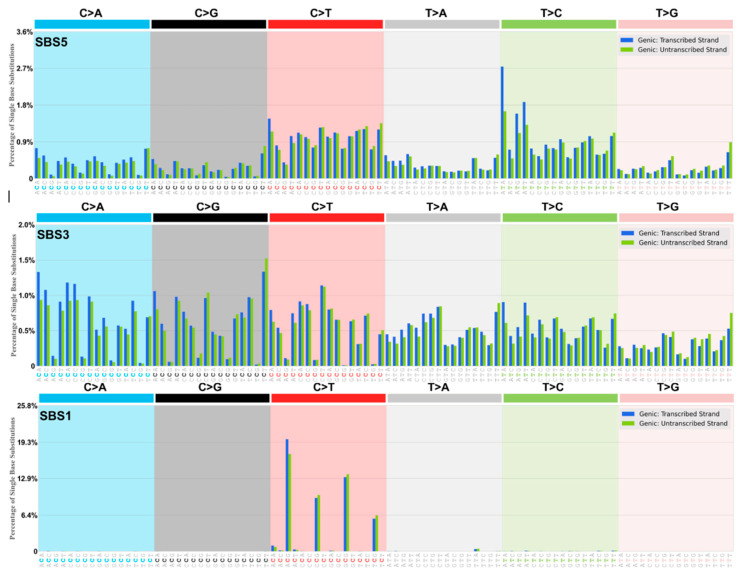
**Transcriptional Strand Asymmetry Profiles SBS5, SBS3 and SBS1**. These profiles are taken directly from the publicly available COSMIC website from Single Base Substitution Mutational Signatures (v3.4 October 2023) at the COSMIC website at https://cancer.sanger.ac.uk/signatures/sbs/ accessed on 15 April 2024 [10,11,12]. URL accessed between 7 and 15 April 2024. Transcriptional stand asymmetry in SBS5 is notable at ACN and ATN trinucleotides, and to a lesser extent in others TCN and TTN (mutated base underlined) where the strong strand bias A>>T and G>>C (Table 1) is now often reversed to T>>A and C>>G when read from the non-transcribed or coding strand. See discussion of Table 1 and Table 2. In SBS3 it is a far flatter profile with patterns of C>A, C>G, C>T broadly elevated across the C-site trinucleotides. A similar flatter profile at T>A, T>C and T>G trinucleotides. See Table 1 for statistical significance of the main profiles. Tables S1–S3 have summaries of the types of mutations observed in different cancer types. For SBS1 the dominant signature is G-to-A exceeding C-to-T systematically across all tissues *p* < 0.001 mainly at ACG motifs; the apparent reverse strand bias in the SBS1 profile at CCG, GCG, TCG motifs is not significant.

**Figure 4:**
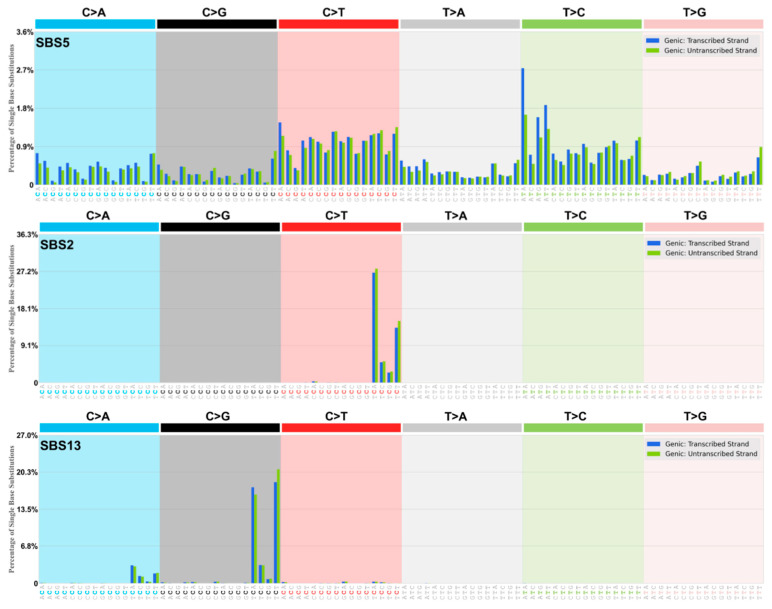
**Transcriptional Strand Asymmetry Profiles SBS5 compared to SBS2, SBS13**. These profiles are taken directly from the publicly available COSMIC website. Single Base Substitution Mutational Signatures (v3.4 October 2023) at the COSMIC website at https://cancer.sanger.ac.uk/signatures/sbs/ accessed on 15 April 2024.

The DRT-paradigm is summarized as a set of “dysregulated Ig SHM-like” strand biased patterns; for example, as observed in the dominant yet ‘flat-like’ COSMIC signature SBS5 (Figure 3 and Figure 4, Table 1 and Table 2). We postulate that the transient assembly (and disassembly) in the cell nucleus of AID-associated Ig-SHM like enzymes and membrane-anchoring factors create a potential “Ig SHM-like Transcription Factory” environment at many sites across the cancer genome and as described by the cell biology work on Transcription Factories by Peter R Cook and associates (reviewed in the context of Ig SHM [53]). The comprehensive genome-wide studies on putative AID-driven Ig-SHM like mutations in the human lymphoblastoid cell line Ramos by David G Schatz and associates are consistent with this view [54]. It would involve the RNA Pol II elongation complex generating a Transcription Bubble at protein coding genes. Such Transcription Bubbles often transiently stall downstream of the transcription start site, to approximately 3 Kb downstream of the TSS [39], allowing mutagenic deaminase action at exposed DNA and RNA substrates at nascent RNA stem-loops [27], the annealed 11 nt RNA:DNA hybrid [35], and the unpaired ssDNA in the displaced NTS. Extreme examples of much longer extended RNA:DNA annealed hybrids would be in R-Loops, particularly evident in cancer genomes at Transcription Replication Fork collisions (TRC) on the same strand [25,26], and R-Loop formation at telomeres [24].

### 2.1. Strong Evidence for DRT Origin of Many SBS Signatures

What follows is a detailed critical analysis of the mechanism of origin of a number of significant SBS signatures in the context of the AID/APOBEC/ADAR deaminase mutational DRT-paradigm. To better understand these analyses, it is advised that the reader refer continuously to the transcriptional strand asymmetry signatures for that SBS at the COSMIC website (e.g., as seen in Figure 3 and Figure 4). Go to “Single Base Substitution (SBS) Signatures” at https://cancer.sanger.ac.uk/signatures/sbs/ (accessed on 15 April 2024), scroll to the SBS signature you select, click that site, go to the chosen “SBSxx Topographical Features” then click “Transcriptional strand asymmetry” to clearly see the histogram plots, as in Figure 3 and Figure 4, i.e., Genic: Transcribed Strand (blue) and Genic: Untranscribed Strand (green), and you can download the Excel files used here to analyze in detail, as we have done in Materials and Methods Section 3.2, e.g., also Supplementary Table S1. A tabular summary of the conclusions and outcomes is displayed in Table 3.

#### 2.1.1. Origins SBS5

The etiology of SBS5 is unknown and is described as “flat” and “Clock-like”, thus age-related. It is the most dominant SBS signature appearing prominently in frequency in all cancer genomes [10,11,12]. There is a general agreement that SBS5 is the result of the accumulation of many somatic mutations over time and cell division cycles arising from the interplay of DNA damage and repair in the broadest meaning of that description [59]. The over-arching feature is the significant transcriptional strand bias at A:T and G:C base pairs displayed by this pan-cancer signature (Table 1 and Table 2).

The somatic mutation pattern for SBS5 is displayed as a “Types of Mutation” pattern in Table 1B in relation to the same pattern, as observed in well-defined experimental somatic mutation assays observed in Germinal Centre B lymphocytes from Peyer’s Patches and spleens of aged or immunized inbred mice and transgenic systems where PCR recombinant artefacts, that blunt strand bias, have been minimized (Table 1A). The data in Table 1B are integrated and pooled across many thousands of sequenced cancer genomes of different cancer tissue types (see Table S1, and strand biases further summarized by cancer type in Table 2). The mutations of A systematically exceed the mutations of T (symbolized as A>>T), and the mutations of G systematically exceed the mutations of C (symbolized as G>>C) at *p* < 0.001. However, within A:T base pairs, the mutations at A-to-C complement T-to-G and *go against this trend*, with T-to-G mutations significantly exceeding A-to-C mutations (*p* < 0.001). This result is consistent across the majority of different cancer tissue types displaying SBS5 signature patterns (Table 2, see Table S1).

Why are these patterns so? Two of the three main sources of deaminase substrates (ssDNA, RNA:DNA hybrids) are associated with the Replication Stress (Lagging strand of the Replication Fork, ssDNA) and Transcriptional Stress (R-Loop generation, ssDNA, RNA:DNA Hybrids). In our view, the third source of deamination substrates are at Stalled Transcription Bubbles, as is the case for the more defined Ig SHM systems providing ssDNA, dsRNA stem-loops, and RNA:DNA hybrid substrates, which together provide substrates for AID/APOBEC C-site and ADAR A site deaminations (Figure 1 and Figure 2). These mutagenic events coupled to TSRT mediated by DNA Polymerase-eta, as for Ig SHM, generate the strong strand biased mutagenesis signal that outweighs the contributions from the other two mentioned sources. Blunting this systematic A>>T strand bias in SBS5 is the systematic strand bias of T-to-G over A-to-C mutations. This strand bias is not evident in the Ig SHM data (Table 1A), but it is the case in the vast majority of different types of cancer genomes with sufficiently large enough mutation numbers to assess significance (Table 2, see Table S1). There are exceptions to this T-to-G strand bias viz. Squamous Cell Carcinoma of the Head and Neck (Head-SCC), and Lung cancer (Lung-SCC).

The strand biased pattern for the origins of T-to-G>A-to-C in the vast majority of cancers is plausibly explained by the DRT-Paradigm by assuming a major role for the modified isomer of uracil in pre-mRNA, pseudouridine (ψ), now behaving like “G” base pairing to mis-incorporate a C in the newly synthesized cDNA transcribed strand (TS) via TSRT opposite ψ in the pre-mRNA. There is much data in the pseudouridine literature consistent with this explanation [17,18,19,20].

What is a plausible explanation for the other less dominant yet reverse strand biases of A<<T, at certain trinucleotides such as TTC, TTT? These are at classic WA site motifs for ADAR A-to-I RNA deamination [60]. One likely explanation is the role of ADAR1 and ADAR2 assisting R-Loop dissolution, particularly at ubiquitous transcription-generated R-Loops at replication fork head-on collisions or TRC [25]. The annealed RNA:DNA hybrid regions are a target for ADAR1 attack for dissolution of R-Loops at telomeres [24]; and, ADAR2 does this in the wider body of the genome at R-Loop TRC sites [25].

Double strand DNA breaks (DSBs) can provoke R-Loop formation [61] and change the general pattern of ADAR2 A-to-I RNA editing that assists their resolution [62] and specifically assists DNA end resection and homologous recombination (HR). These observations are thus consistent with observed genomic mutagenesis in cancer on foci of R-Loops at TRC sites [25].

The almost universal T-to-G strand bias observed in cancer genomes prompts an additional comment. If the suggestion that excessive pseudourinylation (ψ) in cancer transcriptomes is correct, then it warrants further investigation to establish whether or not ψ is a useful pan-cancer biomarker.

#### 2.1.2. Origin SBS1

This “Clock-Like” signature at CpG sites appears in all cancers examined (Figure 3). The formal description and etiology is a “Clock-Like” (i.e., age-related) signature arising due to spontaneous water deamination or enzymatic deamination of the methylated cytosine at NCG (read CpG) sites.

The dominant ACG motif among the C>T trinucleotides harbors approximately 36% of all substitutions within this signature. At ACG, a G-to-A>C-to-T strand bias is evident when mutations are read from NTS (not significant at the other lower incidence CpG motifs, CCG, GCG, TCG). SBS1 is a minor extracted SBS signature. It is a G>>C strand bias component of the global AID/APOBEC driven strand bias at C-sites in the SBS5 signature.

The current biochemical data are inconsistent with SBS1 arising spontaneously by water hydrolysis in vivo [11,12] and is most likely AID or APOBEC deaminase driven when it appears in cancer genomes in vivo. In our view, SBS1 falls under the umbrella of the DRT-Paradigm.

Detailed comparative biochemistry analysis in vitro by Ito et al. [63] of deamination C or mC to uracil or thymine suggest this is an enzyme catalyzed AID/APOBEC deamination signature and that spontaneous water hydrolysis is an unlikely deamination event. Indeed, methylation of C at CpG sites appears to protect cytosines from enzymatic deamination. There is a range of dose dependent activity (“catalytic efficiency”) for C and mC deamination across a range of substrate motifs (see data in figure one in the paper by Ito et al. [63]). These include a TCpG site and ACA, CCA, GCA, TCC, TCT, TCA, CCC motifs. The deamination efficiency against relevant substrates, methylated or not, was compared for cytosine deaminases AID, APOBEC1, APOBEC3A though to APOBEC3H. In all cases unmethylated C-sites were deaminated effectively but at varying dose-dependent efficiencies. What is striking is that *in all cases*, when the same C-centered motif is methylated, substantial reductions, down 51–98% in deamination efficiency, occurred across the range of AID and APOBECs tested. Indeed, APOBEC3H was “inhibited” in its deamination activity the least by cytosine methylation.

Consistent with this view is the finding that SBS1 is depleted across cancer types for multiple histone marks, including H3K9me3 [12]. One speculation is that this is a consequence of excessive methylation of cytosines protecting against AID/APOBEC deamination of 5meC sites in general.

#### 2.1.3. Origin SBS2/SBS13 (See Figure 4)

These are C-site mutations (Box 1) targeted at G:C base pairs [11,12,64]. The designated origin is attributable to aberrant activity of the AID/APOBEC family of cytosine deaminases particularly APOBEC3A, APOBEC3B, APOBEC3H at lagging strands of replication forks under stress (see Supplementary Information Section S2A). They do not display systematic transcriptional strand asymmetry, but they do show replication strand asymmetry with a preference for the lagging strand indicative of unpaired cytosines on the ssDNA substrates at replication forks. It is agreed that the strand bias at G:C base pairs in SBS13 is most likely generated by the translesion repair enzyme REV1 replicating across abasic sites arising from BER removal of uracil (reviewed in the context of Ig SHM [9]). The SBS2/SBS13 signature (Figure 4) appears jointly and to varying degrees of strength in many cancers (24/32 in [11]). It can be considered as a small and defined subset of the global AID/APOBEC and ADAR strand-biased deaminase-based TSRT signatures already discussed for SBS5, using the DRT-Paradigm. This interpretation also assigns causative “AID/APOBEC” activity to the SBS2/SBS13 at the online COSMIC database [11,12], as shown in the summary Table 3.

#### 2.1.4. Origin SBS3

This complex SBS signature (Figure 3, Table 1) appears in a subset of tumors with Defective Homologous Recombination (dHR) Repair of double strand breaks (DSBs) due to genetic deficiency in BRCA1 or BRCA2 genes. It is a complex signature, and many features are not inconsistent with the DRT-paradigm interpretation.

There are strong parallels in the genomic sequencing analysis on in vitro culture of the avian DT40 cell system consistent with the SBS3 profile [65]. Superficially, it appears similar and “flat” to the SBS5 profile, but there are many differences. Clearly, many unrepaired single base substitution lesions, apart from the more serious DSBs, are elevated in HR Defective patients. The patients themselves are surprisingly long lived given the seriousness of the formal HR deficiency, suggesting other DNA repair mechanisms compensate, which suggests back-up RNA-templated DSB repair via DNA repair reverse transcriptases, Pol-eta [49], and thus putatively Pol-theta, which is also a reverse transcriptase [66] via TSRT as already discussed.

At A:T base pairs, the global strand biased A>>T pattern is not significant, although there is a clear strand bias for A-to-G exceeding T-to-C mutations, the prominent strand bias in SBS5. There are many distortions in patterns to the “types of mutations” that are systematic in SBS5 (Table 2), as seen in Table S2. Many of the cancers with this profile may also have potential “smoking” adduct or etiology for those mutation patterns; or accumulated endogenous adducts on G, and maybe A as well (Table S2). In this regard, BRCA1 deficient DT40 cells display 53BP1 dependent translesion Y family involvement of Pol-eta, Pol-kappa. This dependency on specific base substitution mutations on Pol-eta, Pol-kappa for translesion synthesis [67] is very interesting given that human Y family polymerases eta, kappa, and iota are all known to display reverse transcriptase activity [50,68,69].

Final caveats on the putative involvement of TSRT in the generation of some of the SBS3 signature, particularly at purines G and A are noted. This strand bias could also result from exogenous sources such as tobacco smoking. It could also arise via spontaneous endogenous bulky adducts on G and A, thus conventional Transcription Coupled Repair (TCR) [70,71,72], making detected mutations on the NTS exceed those on the TS, as is clear in the “tobacco smoking” signature of SBS4.

However, ROS-generated 8oxoG modifications in nascent pre-mRNA cannot be ruled out as a primary source of excessive strand biased G-to-T mutations (see below Section 2.1.16).

Further, SBS3 is a minor signature in most BRCA1/2 deficient cancers, except in Breast-Cancer, ESCC, Ovary-AdenoCA, Panc-AdenoCA, and Stomach-AdenoCA (Table S2). It is conceivable within the SBS3 profile, that there is also some endogenous ADAR A-to-I damage at A-sites in pre-mRNA, at uracil isomerization in pre-mRNA (ψ), and at Transcription Bubbles, thus making an expected contribution of A-to-G>T-to-C. The global G>>C strand bias is also prominent and may suggest the involvement of Pol-eta (or putative Pol-theta) TSRT repair, as discussed for SBS5. This appears particularly the case for Breast-Cancer (Table S2).

In summary, a number of processes generating strand bias effects appear to contribute to the SBS3 profile, including the bulky adduct clearance of adducted Gs and As by conventional TCR, AID/APOBEC, and ADAR deaminase-driven reverse transcriptase-coupled processes involving TSRT and back up RNA-HR reverse transcriptase-mediated DSB repair.

#### 2.1.5. Origin SBS4

This is the classic “tobacco smoking” signature. It occurs mainly at G:C base pairs but also at lower frequency at A:T base pairs. The undisputed conventional explanation is that the SBS4 transcriptional strand biases at G:C and A:T base pairs are caused by preferential bulky adduct clearance of adducted Gs and As on the transcribed strand by conventional transcription coupled repair (TCR). This signature has long been considered to be diagnostic of DNA mutagenic damage associated with tobacco smoking [70,71,72].

#### 2.1.6. Origin SBS6

The proposed etiology for this signature is defective DNA mismatch repair (dMMR) with bias to the leading strand at replication forks [12] and is found in microsatellite unstable tumors. It appears at significantly low incidence in a very small number of cancers (Liver-HCC, Lymph-BNHL, Panc-AdenoCA, Uterus-AdenoCA [11]. The prominent apparent reversal of G-to-A over C-to-T strand bias (as SBS5, Table 1 and Table 2) at some motifs (CCG, GCG, GCT, TCG, but not ACA, ACG) is similar to patterns at the same motifs in SBS1 (Figure 3). None of these apparent strand biases reach significance, and the numbers of mutations are small. SBS6 is considered a small subset of the AID/APOBEC deaminase driven C-site signature of SBS5 (The DRT-Paradigm).

#### 2.1.7. Origin SBS7a, SBS7b and SBS7c, SBS7d

These have been attributed to exogenous UV exposure observed in Skin-Melanoma genomes [11,12]. Many of the component transcriptional strand biased signatures at both G:C and A:T base pairs can be plausibly understood within the frame of the DRT-paradigm.

The main G:C base pairs targeted mutations in Skin-Melanoma are caused by the formation of cyclobutene pyrimidine dimers (CPD) in DNA. This is a significant damage lesion in the DNA helix blocking transcription and replication passage. It is responsible for >95% of all C>T signature mutations (of C-to-T and G-to-A) in Skin-Melanoma genomes. These numbers and statistics for strand biases at G:C and A:T base pairs are summarized in Table S3 (harvested from https://cancer.sanger.ac.uk/signatures/sbs/ accessed on 15 April 2024). It can involve a two-step process in human cells involving cytosine deamination (C-to-U) at certain motifs then error-free polymerase bypass repair [73]. The UVB exposure causes cyclobutene pyrimidine dimers (of adjacent pyrimidines written as C=C, T=C). The authors tested their hypothesis that largely confirms this alternative mechanism.

The main assumption in Jin et al. [73] is that the cytosines in the CPDs are deaminated by spontaneous processes to form uracil, which are then faithfully replicated by Y family translesion DNA polymerase eta, and thus incorporate adenines across the deaminated, or uracil-containing CPDs. The resulting mutations in the tri-nucleotide spectrum broadly matches SBS7 (SBS7a,b), which is a very good confirmation of their alternative explanation for adjacent T-T sites appearing at T=C sites within CPDs after UVB exposure and CPD repair.

This is a reasonable explanation apart from the assumption that the recovery and repair process on UVB exposure involves non-catalytic or spontaneous cytosine deamination. Our doubts about this assumption are supported by the experimental method the authors employed [73]. We propose an alternate explanation based on their method of UVB exposure and recovery prior to sequencing the products. The authors irradiated human fibroblast cells with UVB and harvested them 24 and 48 h later to allow time for deamination [73]. In our view, that period of 24–48 h for deamination to occur is the alternative key to understanding these data. This time interval is consistent with the immediacy and time course of a cellular Innate Immune response. It is indeed plenty of time for the Innate Immune response to be marshaled and assembled following this quite powerful attack on the integrity of the cell, particularly the DNA damaged genome. In our opinion, a cellular Innate Immune response is unavoidable.

Thus, our alternative contention is that sunlight UVB damage, such as CPD lesions across the genome, particularly in coding regions, can excite an Interferon Stimulated Gene-dependent Innate Immune response, which includes APOBEC and ADAR activation [29]. This itself is also likely to activate expression of the DNA damage regulator TP53 that is known to coordinate expression of APOBEC3 family genes [42]. Thus, APOBEC3G [74], APOBEC3B, and APOBEC3A at least can expect to be activated [75,76,77], causing expected collateral genomic damage via DNA deaminations [3,5] particularly in melanoma [78] and thus cancer pre-mutations—via C-to-U mutations at T=C and C=C cyclobutane pyrimidine dimers, involving error free DNA direct copying damage repair by DNA Polymerase eta.

In our view, SBS7a/b is a cancer mutation signature involving both active deaminase-driven cytosine deamination coupled at least to translesion DNA repair synthesis involving DNA polymerase eta.

However, not explained is the strong transcriptional reverse strand bias (in relation to SBS5) of G<<C (i.e., C-to-T>G-to-A) in both SBS7a, SBS7b, and at a far lower level of T site mutations, which exceed A site mutations (Table S3). How do these strong and highly significant transcriptional strand biases in the C>T and T>C tri-nucleotide spectral patterns arise (without replication strand bias)?

Plausible explanations that fit the data are in two parts:

1. SBS7a, SBS7b: The strong transcriptional strand bias at TpC-sites first involves the C-to-U deamination step, as shown in Jin et al. [73]. Pol-eta may well be involved in the error-free repair. However, CPDs in the cell genome would also be expected invoke a strong conventional TCR process [70]—involving NER-TCR—directed at the preferential repair of the template or transcribed strand (TS) for RNA Pol II transcription leaving an excess of unrepaired C-to-T mutations on the displaced non-transcribed strand (NTS). CPDs are akin to obstructive bulky adducts on the template strand, which would be cleared preferentially, as shown earlier for bulky adducts of purines [71,72], as observed in SBS4 (tobacco smoking).

In our opinion the extreme strand biases at G:C base pairs in the SBS7a and SBS7b profiles result from conventional TCR.

2. SBS7c, SBS7d: These mutation levels are <5% of all mutations in Skin-Melanoma genomes. In our opinion the reverse strand biases e.g., T-to-C far exceeding A-to-G require a different explanation as it involves specific mutations at A:T base pairs. The most plausible in progressing malignant melanomas would be the ubiquitous and putative large number of R Loop-Replication Fork conflicts [25], as already discussed to explain similar reversals in strand biases in SBS5. Thus, we invoke ADAR1/2 involvement in the Inosine modification of adenine bases in the DNA moiety of the long annealed RNA:DNA hybrids at R Loops. This would then assist in the release of the pre-mRNA and its degradation, thus dissolution of R Loops as discussed above. The extreme T-to-C strand bias over A-to-G follows replication of the unrepaired Inosine (Hypoxanthine) in the DNA at the collapsed R Loop site. Given Wobble Base pairing off template Hypoxanthine, other possible extreme strand biased signatures at T appearing on the NTS would be T-to-A viz. at TTT trinucleotide motifs (AAA on the TS).

We support both of these explanations, although different, as they are economical on basic assumptions, and provide plausible explanations for the intriguing strand biases of SBS7. Together, both explanations are consistent with AID/APOBEC and ADAR deaminations as initiators and drivers of DNA damage in melanoma progression post UV exposure. They are, thus, part of the DRT-Paradigm we employ in our analytical approach to understand the generation of SBS strand bias signatures.

It is noted that the strong presence of a T-to-G>A-to-C strand bias, which we have speculated, is caused by endogenous pseudouridination (ψ) of uracil in cancer transcriptomes (Table S3), and now coupled to TSRT (Pol-eta, Pol-theta), as discussed earlier as the base mispair outcome of the RNA modifications appears in genomic DNA.

#### 2.1.8. Origin SBS8

Classed as of “unknown etiology”, it is similar to the signature of alkylation of G and A by methyl methanesulfonate exposure in avian DT40 cells [67]. However, both C-site and A-site Transcriptional Strand Asymmetry is noted at G:C and A:T base pairs. A plausible origin is exposure to alkylating agents [31] (endogenous or exogenous?) and the strand biased profiles are suggestive of bulky adducts of G, A, and T resulting in G-to-T, A-to-T, and T-to-A excesses on the non-transcribed strand via conventional TCR with preferentially targeting of the transcribed strand [70], as originally described for bulky adducts of tobacco smoking c.f SBS 4 [71,72].

#### 2.1.9. Origins SBS9

This signature is classed [11] as “In part, polymerase eta activity”. It is classed in Box 1 as a C-site plus A-site more or less balanced “Ig-SHM-like” (AID/APOBEC/ADAR driven transcriptional strand biased signatures with some TSRT and some Hx in DNA after R Loops have collapsed and replicated (Table 3).

It appears primarily in lymphocytic and lymphoma tumors (Lymph-BNHL, Lymph-CLL). We are genuinely puzzled by this categorization involving Pol-eta activity. In our opinion, DNA Polymerase eta (and theta) can be involved in target site reverse transcription (TSRT) in the strand biased fixation of RNA mutations in DNA as in Ig SHM (Figure 2). Most of the mutations are at A:T base pairs in the T>C and T>G tri-nucleotide components of the SBS9 profile (https://cancer.sanger.ac.uk/signatures/sbs/ accessed on 15 April 2024). Parts of the patterns are interesting with systematic strand bias to the NTS of T-to-A, T-to-C, and T-to-G. These are understandable under the DRT-Paradigm given previous listed analyses (SBS5), yet it involves Hx in DNA at collapsed R Loops.

First, for T-to-C strand bias to the NTS. In our view, this would plausibly involve ADAR1/2 A-to-I editing of the DNA of the annealed RNA:DNA hybrid at R Loops, as they are collapsed and dissolved, in rapidly proliferating lymphocyte cancers. Then, the unrepaired template Hypoxanthine is copied as T-to-C into synthesis of the NTS on replication as discussed (SBS5).

Second, the origin of the T-to-G strand bias could also plausibly involve pseudouridine (ψ) modifications in RNA as discussed and TSRT fixation of T-to-G mispaired mutations in the genome via DNA Polymerase eta acting in its reverse transcriptase repair mode (TSRT).

The main features in SBS9 are understandable from first principles and DRT model assumptions (AID/APOBEC and ADAR deaminations coupled to TSRT). However, also note the analysis [12], where the strong replication strand bias with enrichment of mutations on the leading strand is attributed to the infidelity of polymerase eta.

#### 2.1.10. Origin SBS10a, SBS10b, SBS14

These signatures are associated with replicative DNA polymerase epsilon or *POLE* gene mutations—with or without dMMR. A mutation in the *POLE* gene is associated with faulty polymerase proofreading. There is no reason to dispute the attributed origins of these very minor signatures that appear in Colorectal-AdenoCA, Uterus-AdenoCA, and Liver-HCC as a consequence of *POLE* mutation(s), with or without MMR deficiency. However, Otlu et al. [12] attributes the strong replication strand bias with enrichment of mutations on the leading strand to the defective activity of polymerases, DNA polymerase epsilon (*POLE*) and polymerase delta (*POLD1*).

#### 2.1.11. Origin SBS11

There is no reason to qualify the origins of SBS11, as it is associated with Temozolomide treatment. It is a minor yet distinctive signature in CNS-GBM and Panc-Endocrine tumors. The systematic transcriptional strand bias of G-to-A mutations exceeding T-to-C mutations at many C-site motifs (ACC, ACT, GCC, GCG, GCT, TCC) suggests the involvement of AID/APOBEC deamination coupled to TSRT via Pol-eta (or Pol-theta). Thus, a cytosine deaminase explanation at Transcription Bubbles coupled to genomic fixation via TSRT is plausible. The DRT-Paradigm is useful to understand the transcriptional strand bias features of SBS11.

#### 2.1.12. Origin SBS12

This is one of the most interesting signatures in the SBS collection. It is of “Unknown” etiology and dominates Liver hepatocellular carcinoma (HCC) genomes (see Table 2, Table 3 and Table S1). It is largely focused on A:T base pairs, with lower-level mutations at G:C base pairs. The notable feature is the extreme strand bias of A-to-G mutations strongly exceeding T-to-C mutation on the NTS. A plausible interpretation is that this is caused by the oncogenic tumor promoting activity of high ADAR1 expression in such cancers [79], as discussed elsewhere [7,80]. Others [81], including curators at the COSMIC site, suggest this is an example of an unknown etiology involving Transcription Coupled Damage (TCD) causing lesions at adenines on the (displaced) NTS at Transcription Bubbles. However, in the context of the DRT model (Figure 1a), this is a good, although an extreme example of transcription-coupled ADAR1-mediated A-to-I deamination of nascent pre-mRNA stem-loops [27] followed by TSRT at Stalled Transcription Bubbles then fixing the pre-mRNA A-to-I mutations in DNA. This is the most plausible cause of the extreme strand bias of A-to-G mutations over T-to-C, as read on the NTS. The stand-out features of SBS12 are thus understandable from first principles and foundation assumptions of the AID/APOBEC and ADAR deamination paradigm coupled to TSRT involving the RT activity Pol-eta at least, and/or the putative RT activity of Pol-theta. That is, the DRT-Paradigm.

#### 2.1.13. Origin SBS15

This signature of “Defective DNA mismatch repair” (dMMR) displays features of the DRT-paradigm. It is evident at low level in Biliary-AdenoCA, Colorectalk-AdenoCA, Stomach-AdenoCA, Uterus-AdenoCA [11]. At the COSMIC site (ver3.4), ESCC displays the signature prominently. It is focused at G:C base pairs for the C>T set of trinucleotide motifs, particularly GCG, but is also evident at GCA, GCC, and GCT motifs. These are key features of core RCN AID deaminase motifs (typically WRCG/W). What is striking about SBS15 is the complete lack of Transcriptional strand asymmetry, see https://cancer.sanger.ac.uk/signatures/sbs/sbs15/#transcriptional-strand-asymmetry (accessed on 15 April 2024). A plausible explanation is that defects in the mismatch repair MSH2-MSH6 heterodimer activity may not sufficiently recruit DNA Polymerase eta to AID-mediated C-to-U DNA lesion sites (thus poor TSRT). Such a deficit has been established in a Ig SHM system in vitro by Patricia J Gearhart and colleagues [82]. Thus, the DRT-Paradigm allows us to better understand the lack of transcriptional asymmetry in SBS15.

#### 2.1.14. Origin SBS16

This signature is of “Unknown” etiology, yet it can be plausibly attributed to “Alcohol consumption” on current observations, and mechanistically to what has been termed Transcription Coupled Damage [12,81]. It is evident in Head-SCC and Liver-HCC [11,12]. At the COSMIC site (ver3.4), ESCC and Liver-HCC display this strong A>>T strand biased signature at A:T base pairs prominently (https://cancer.sanger.ac.uk/signatures/sbs/sbs16/#transcriptional-strand-asymmetry (accessed on 15 April 2024)).

SBS16 is, thus, an A:T bp-focused signature at ATA, ATG, ATT motifs, which are core WA motifs for both ADAR1 mediated A-to-I pre-mRNA modifications [60] and indeed DNA Polymerase eta [83,84] during Ig SHM in vivo [27]. The strand biased mechanisms highlighted in Figure 1 apply. As with SBS12, the SBS16 signature is, therefore, understandable from first principles and foundation assumptions of the AID/APOBEC and ADAR deamination paradigm coupled to TSRT, involving the RT activity Pol-eta at least, and/or the putative alternate RT activity of Pol-theta, that is the DRT-Paradigm. See comments Table 3.

#### 2.1.15. Origin SBS17a, SBS17b

This signature is also of “Unknown” etiology, and it appears in many cancer genomes but particularly with high somatic mutation numbers in Eso-AdenoCA, Stomach-AdenoCA and is A:T bp focused. In SBS17a, the reverse strand bias of A-to-G<T-to-C on NTS is significant (*p* < 0.001). In SBS17b, the strand bias of T-to-G>A-to-C at main motifs CTT, GTT, and TTT is also systematically significant (*p* < 0.001).

The explanations under the DRT-Paradigm for these different transcriptional strand biases are in two parts.

In SBS17a, these strand biased patterns are consistent with ADAR-mediated A-to-I creating hypoxanthine in the DNA moiety of long annealed RNA:DNA hybrids at R Loops in these progressing cancers. On ADAR assisted dissolution and degradation pre-mRNA, it can lead to unrepaired hypoxanthine in TS DNA being replicated to produce excess T-to-C (and Wobble Base pairing producing the alternative T-to-A) mutations on the NTS.

In SBS17b, while a contribution from Wobble Base pairing at Hypoxanthine at R Loop dissolution may contribute to the excess in T-to-G over A-to-C, putative pseudouridinylation (ψ) of nascent pre-mRNA, as speculated previously at Stalled Transcription Bubbles followed by TSRT would also contribute to this pancancer signature (see discussion Section 2.1.1 SBS5).

#### 2.1.16. Origin SBS18

This signature, in many cancer genomes, is putatively caused both by the Innate Immune Response to infections and internal cellular stress and DNA damage involving reactive oxygen species (ROS)—acting to oxidize nucleic acids particularly Guanines causing G-to-T mutations in DNA as a consequence of 8oxoG formation. Thus, the strong mutation profile signature of SBS18 is focused on G:C base pairs and most dominantly in C>A trinucleotides. There are two striking features.

The first is the transcriptional strand bias of G-to-T mutations exceeding C-to-A on the NTS. This is particularly evident in ACA, ACC, ACT, CCA, GCT, GCA, GCT, TCA, TCC motifs. In different cancer types with large numbers of mutations the strand bias is very significant in Breast-Cancer, Colorect-AdenoCA, ESCC, Eso-AdenoCA, Stomach-AdenoCA (*p* < 0.001). This is a striking result not in the least because the observation conflicts strongly with known oxidative DNA base damage studies in mammalian cells. Thus, Thorslund et al. [85] investigated defined oxidative DNA base damage exposure of Chinese hamster ovary fibroblast cells in culture. In contrast to mitochondria, they report that 8oxoG is repaired equally on both DNA strands without strand bias. This is expected as 8oxoG modifications are not considered bulky adducted modifications and can be replicated easily or presumably reverse transcribed.

Why do the SBS18 ROS signatures in many cancer genomes in vivo display strong G-to-T over C-to-A strand bias? This is reminiscent of the known similar bulky adduct-induced strand biases caused at Gs in lung cancer mutational hotspots in the TP53 gene on exposure to Benzo[a]pyrene adducts, and their slower removal from the TS [71,72] the now classic strand-biased outcome of Transcription Coupled Repair, as discussed for SBS4 [70].

An answer that fits the transcriptional asymmetry data assumes oxidative RNA damage in nascent pre-mRNA at Stalled Transcription Bubbles, as specifically speculated on earlier [13,14] based on the published RNA oxidative damage studies of Wu and Li [21]. Thus, in this scenario, strong strand biases of the type G-to-T exceeding C-to-A on the NTS can also, in theory, be generated by ROS stress first as RNA modifications (8oxoG), which are converted to excessive strand biased G-to-T mutations via TSRT and reverse transcriptase functions of Pol-eta (or putatively Pol-theta).

The DRT-Paradigm, thus, allows a plausible understanding of these simple base modified strand biases now appearing in genomic DNA of cancer cells.

The second and overlooked feature of SBS18, is the significant 5′ preference for G-to-T mutations. Thus, on average, the incidence of G-to-T mutations (8oxoG) at WG sites is four times more frequent than at SG sites (S is G or C). This has similarities to accessibility of ADAR deaminases to the A-site at WA motifs in dsRNA [60]. It appears that oxidation at the 8 position of G via ROS follows similar biochemistry.

The transcriptional strand asymmetry signature of SBS18 is understandable in part in terms of the DRT-paradigm.

#### 2.1.17. Origin SBS19

The etiology of this signature is “Unknown”. It appears as a minor signature in CNS-PiloAstro, Liver-HCC and Myeloid-MDS/MPN tumor genomes. The striking transcriptional strand asymmetry profile shows it is almost a pure G-to-A>C-to-T strand biased signature. Again, a signature that is best understood under the DRT-Paradigm.

#### 2.1.18. Origin SBS84, SBS85

In Otlu et al. [12] these are assigned as “AID-associated signatures SBS84 and SBS85”. This implies off-target Ig SHM-like mutagenesis across the cancer genome. The reverse transcriptional strand-bias is significant, particularly at A:T base pairs T-to-C> A-to-G and T-to-A>A-to-T. This suggests R Loop targeting and unrepaired hypoxanthines in DNA in the transcribed strand after nascent RNA release and degradation. The key points on this reverse strand biased feature have been made viz. Section 2.1.1 SBS5 (and Table 3).

#### 2.1.19. SBS Signatures SBS20 Through SBS 44 (as [11])

This paper will not critically evaluate these signatures here, as the main conceptional and interpretation points concerning the DRT-Paradigm have been established, in our opinion, by the above analyses. These additional signatures are all minor mutation patterns apart from SBS40 (“Unknown” etiology, yet it appears much like SBS5). Some are repetitive subsets of other established signatures. Many have no known causes. However, many also have no topographical “Transcriptional strand asymmetry” assigned at time of writing, e.g., SBS40.

## 3. Materials and Methods

### 3.1. Cancer Genome Sequence Source Data

All somatic mutation data in sequenced cancer genomes was sourced between 7 and 15 April 2024 at the Catalogue Of Somatic Mutations In Cancer (COSMIC) online site (v3.4) at https://cancer.sanger.ac.uk/signatures/sbs/ (accessed on 15 April 2024).

### 3.2. Conversion of Transcriptional Strand Asymmetry SBS Somatic Mutation Numbers at the COSMIC Site to “Types of Mutation” Tables

We converted the COSMIC (v3.4) presentation of “Transcriptional strand asymmetry” files (under Topographical features) to a more familiar format for viewing strand biases by the construction of “types of mutations” tables typical of published Ig SHM analyses at Ig loci as Table 1 [7]. In such tables, all 12 types of somatic mutations are read from the coding strand, or in the present terminology, on the displaced non-transcribed strand generated by RNA Polymerase II Transcription Bubbles, NTS.

The analytical steps for conversion of a SBS signature is as follows:

#### 3.2.1. Step One of Conversion

We converted the “Transcriptional strand asymmetry” data for each SBS signature for accumulated real mutations at each of the pyrimidine-centered trinucleotide motifs (C>A, C>G, C>T, T>A, T>C, T>G) on the “transcribed” strand and left unaltered the real mutations on the “untranscribed” strand. For example, at G:C base pairs, real mutations of C>A on the “transcribed strand” are now read as G>T mutations on the “untranscribed strand” (and numbers of real C>A on “untranscribed strand” left unaltered); similarly real mutations of C>T on the “transcribed strand” are now read as G>A on the “untranscribed strand”; and similarly for A:T base pairs real mutations of T>C on the “transcribed strand” are read as A>G mutations on the “untranscribed strand (and T>C real mutations on the “untranscribed strand” left unaltered). This exercise is repeated for each pyrimidine centered trinucleotide motif.

#### 3.2.2. Step Two of Conversion

**Figure 5:**
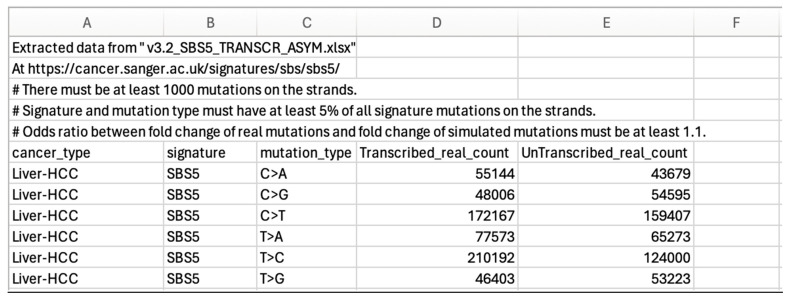
**Conversion text file to Excel file**. SBS5 Liver-HCC text file “v3.2_SBS5_TRANSCR_ASYM.xlsx”. https://cancer.sanger.ac.uk/signatures/sbs/sbs5/ (accessed on 15 April 2024).

This is illustrated for SBS5 Liver-HCC. In the first step (Figure 5), the downloaded text file that has been converted to an excel file is “v3.2_SBS5_TRANSCR_ASYM.xlsx” and SBS5 Liver-HCC data extracted as shown in the screen shot below.

#### 3.2.3. Step Three of Conversion

**Figure 6:**
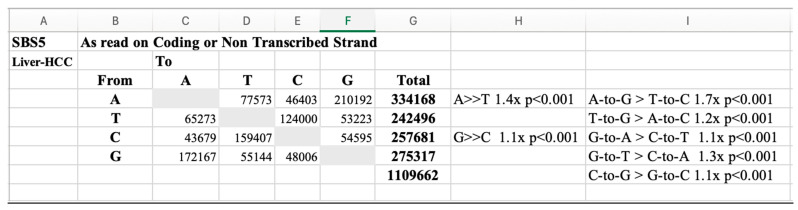
**Conversion text file to Excel file**. Conversion of real mutations to a “Types of Mutation” table.

The next step (Figure 6) is the conversion of the real mutations to a “Types of Mutation” table, as presented in Supplementary Table S1, where strand biases are easily visible particularly at large N values and Chi-Squared tests (2 × 2) performed directly and p level recorded as NS (*p* > 0.05), then significance level at *p* < 0.05, *p* < 0.01, or *p* < 0.001) tabulated as in screen shot below (From Supplementary Table S1).

## 4. Summary and Conclusions

The focus of our analysis is applied to understanding the likely origin of key COSMIC SBS signatures, using the DRT paradigm (Table 3) [11,12]. Most signatures analyzed can be understood within the context of the DRT model (Figure 1). Most implicate roles for deamination of cytosines or adenosines in DNA or RNA substrates and include coupling to a reverse transcription step. This interpretation and re-evaluation of the base mechanisms driving somatic mutagenesis is consistent with the original hypothesis that cancer genomes display a “dysregulated AID/APOBEC Ig SHM-like signature” coupled with an ADAR deaminase RNA editing signature and reverse transcription [3,5,7,8,13,14].

As we continue to construct our knowledge of the likely source(s) of different mutational signatures in cancers, our main conclusion here is that we can use the DRT model to provide a more detailed molecular explanation of the roles of the mutagenic homologous families of deaminases during oncogenesis. The SBS suite of signatures is a significant advance, yet the next steps may need even more complex predictive molecular models than the DRT model posited here, to be able to identify and assign causation to the many genomic signature differences observed between different cancers and individuals within a cohort.

The main advantage of viewing the molecular processes involved through the DRT prism is two-fold: First, we need to do much more work to better understand the transcription-linked molecular processes contributing to the spectra of mutagenic signatures arising during oncogenesis. Second, the DRT model, and its molecular processes, can potentially lead to the future development of more therapeutically precise predictions for individual patients, and for the personalization of the patient clinical care treatment path.

## Data Availability

All data analyzed in this paper are provided here or in Supplementary File and primary mutation data available online at the COSMIC website.

## Supplementary Materials

The following supporting information can be downloaded at: https://www.mdpi.com/article/10.3390/ijms26030989/s1. References [86,87,88,89,90,91,92,93,94,95,96,97,98,99,100,101,102,103,104,105,106,107,108,109,110,111,112,113,114] are cited in Supplementary Materials.

## Abbreviations

(Full list in Supplementary file): **A>>T**—mutations in DNA at A exceed mutations at T; **A-to-I**—adenosine to inosine RNA editing of adenosine preferentially at **WA** sites—in DNA the product is called hypoxanthine (**Hx**); **ADAR**—adenosine deaminase acting on RNA causing **A-to-I** modifications (read as A-to-G mutations) ; **APOBEC**—apolipoprotein B mRNA-editing, catalytic polypeptide; **AID**—activation-induced cytidine deaminase, a member of APOBEC family of cytosine deaminases, that initiates via **C-to-U** DNA editing (or **C-to-T** mutations after unrepaired replication) in single stranded DNA both in rearranged immunoglobulin (**Ig**) variable gene (**V[D]J**) somatic hypermutation (**SHM**) and heavy chain isotype class switch recombination (**CSR**); **G>>C**—mutations in DNA at G exceed mutations at C; **C-to-U**, cytosine to uracil mutations at appropriate AID (**WRCN**) and APOBEC3 family protein deaminase motifs at e.g., **TCN, CCG**, read as **C-to-T** cytosine to thymine at 5meCpG site motifs; **TSM**, targeted somatic mutation in codon context; **DRT** model, deaminase-driven reverse transcriptase mutagenesis; **TSRT**—target site reverse transcription; **N**, any base; **R**, purine base A and G; **S**, strong base pairing G and C; **W**, weak base pairing A and T/U.
