# Supplementary material for: Deaminase-Driven Reverse Transcription Mutagenesis in Oncogenesis: Critical Analysis of Transcriptional Strand Asymmetries of Single Base Substitution Signatures
Source: Int J Mol Sci. 2025 Jan 24;26(3):989. doi: 10.3390/ijms26030989 (PMC11817618; doi:10.3390/ijms26030989)
Supplement: Supplementary file 1 [file ijms-26-00989-s001.zip › ijms-3351625-supplementary.pdf]

Supplementary File

Deaminase-driven Reverse Transcription Mutagenesis in Oncogenesis : Critical analysis of transcriptional strand asymmetries of single base substitution (signatures

by Edward J. Steele and Robyn A. Lindley

Table of Contents

Section 1: Abbreviations and definitions	p.1-2
Section 2: Aim, structure, background of analysis	p.3-7
Section 3: Supplementary Tables S1, S2, S3, S4	p.8-17
References	p.17-21

Section 1: Abbreviations and Definitions

Note : Base v Nucleotide nomenclature. Commonly used names and correct usage

A - Adenine is the purine base, adenosine is the base covalently bound as a nucleotide to ribose (RNA) or deoxyribose (DNA), and one or more phosphate groups

T- Thymine is the pyrimidine base, thymidine is the base covalently bound as a nucleotide to deoxyribose (DNA), and one or more phosphate groups (polynucleotide).

C- Cytosine is the pyrimidine base, cytidine is the base covalently bound as a nucleotide to ribose (RNA) or deoxyribose (DNA) and one or more phosphate groups (polynucleotide).

CPD - cyclobutane pyrimidine dimers

G - Guanine is the purine base, guanosine is the base covalently bound as a nucleotide to ribose (RNA) or deoxyribose (DNA) and one or more phosphate groups (polynucleotide).

A - adenine base in RNA or DNA

A-to-G > T-to-C - in a 'Types of Mutation Table' indicates frequency A-to-G mutations exceeds its Watson Crick complement T-to-C mutations on the coding or NTS. this symbolism applies to all Watson-Crick complements viz. G-to-A > C-to-T and so on. If the reverse it is symbolised as A-to-G < T-to-C and so on.

A>>T - mutations in DNA at A exceed mutations at T

A-to-I - adenosine to inosine RNA editing of adenosine, in DNA the product is called hypoxanthine

ADAR - adenosine deaminase acting on RNA

APOBEC - apolipoprotein B mRNA-editing, catalytic polypeptide

AID - activation-induced cytidine deaminase, a member of APOBEC family of cytosine deaminases

APE - apurinic/apyrimidinic endonuclease, cleavage at abasic sites.

C - cytosine base in RNA and DNA

bp - base pair

COSMIC - catalogue of somatic mutations in cancer at (https://cancer.sanger.ac.uk/cosmic/signatures)

BER - base excision repair

cDNA - complementary DNA copied off RNA template by reverse transcription

DRT - Paradigm - Deaminase-Driven Reverse Transcriptase Paradigm, principles and foundation assumptions of the AID/APOBEC and ADAR deamination paradigm coupled to TSRT involving primarily the RT activity of DNA repair Polymerase -eta (η) and/or as back up the putative RT activity of DNA repair Polymerase - theta (θ)

DSB - double strand break in DNA helix, often by exogenous ionising radiation but can involve endogenous sources

EXO1 - exonuclease 1

G - guanine base in RNA or DNA

HR - homologous recombination repair

Hx -hypoxanthine, inosine in DNA arising from deamination of adenine

I - inosine in RNA arising from deamination of adenosine

G>>C - mutations in DNA at G exceed mutations at C

Ig - immunoglobulin
MMR - mismatch repair
MSH2- MutS homologue 2
MSH6 - MutS homologue 6
MSH2-MSH6 - MMR heterodimer
N- any base A,T,C,G or U
NER - nucleotide excision repair
NGS - next generation sequencing
NTS- displaced non-transcribed strand at site of active Transcription
3'-OH - 3'-hydroxyl
PCNA - proliferating cell nuclear antigen
PCR - polymerase chain reaction
Pseudouridine (ψ) - an isomer of uracil
R - purine base A and G
RF- replication fork
RT- reverse transcriptase or reverse transcription RNA-HR- RNA templated homologous recombination repair
SBS- single base substitution
SHM- somatic hypermutation
TCD - transcription coupled damage
TCR- transcription coupled repair
TP53 - gene for tumour suppressor protein p53
TSS- transcription start site
T- thymine base in DNA
TRC- Transcription Replication Fork conflicts on same DNA strand
TS- transcribed or template strand at site of active transcription
TSRT - target site reverse transcription
U- uracil pyrimidine base in RNA, can appear in DNA following deamination of cytosine, base pairs with A
UNG- uracil N-glycosylase
S- strong base pairing, G and C
W- weak base pair A and T/U
Y - pyrimidine base C and T/U

Section 2

Aim and structure of the analysis

The aim of this paper is to assist clinical oncology and research laboratories with alternative genetic understanding of next generation sequencing (NGS) data from cancer biopsies. We therefore focus on the molecular processes behind the genesis of deaminase-mediated somatic mutations that can be observed in NGS sequenced cancer genomes, both whole exomes (WES) and whole genomes (WGS).

It is an in-depth critical comparative analysis of the widely cited Sanger-Welcome Trust COSMIC SBS cancer mutation signatures in relation to the alternative deaminase-mediated interpretation outlined in the paper (DRT-Paradigm). An alternative explanation is offered of the origin of SBS signatures widely cited in the clinical oncology and research literature such as SBS1, SBS2/SBS13, SBS3, SBS5, SBS6, SBS19, SBS12, SBS16, SBS17a, SBS17b, SBS21, SBS9. A summary of the COSMIC SBS signatures by C-site and A-site category is shown in Box 1. The target site reverse transcriptase (TSRT) process is applied as an explanation of transcriptional strand biased mutation patterns at Ig loci (Table 1 undergoing somatic hypermutation as shown in Figure 1).

Rhetorical questions and issues in advance and some tentative answers

When this systematic analysis began, the DRT focus posed many rhetorical questions. Thus SBS2/SBS13 are the only two AID/APOBEC mutation signatures formerly acknowledged as 'APOBEC activity'. Is this conclusion correct? A further question: Why is SBS5 the *dominant* signature in all cancers analysed? Indeed, why does SBS5 display significant A>>T and G>>C strand bias in the face of numerous expected countervailing processes that should blunt further this dominant strand bias signature e.g. numerous ssDNA substrates under Replicative Stress on the Lagging strands at Replication Forks, DNA substrates in R-Loops both in RNA:DNA hybrids and ssDNA substrates in the displaced NTS (R loops are ubiquitous in rapidly proliferating cancer genomes). What is the plausible origin of one particular countervailing strand biased signature, T-to-G, that stands out in all cancer genomes examined? This signature has previously gone unrecognised but is now brought into focus by the DRT-Paradigm.

It may also be asked: Why is the less frequent BRCA1/2 Deficiency signature SBS3, indicative of Homologous Recombination (HR) deficiency, also dominantly strand biased in the same direction at many tri-nucleotide motifs as SBS5? Are the processes underlying both transcriptional strand biases in SBS5 and SBS3 the same in both cases? In contrast, in Lung and related Esophageal cancers (SBS4) associated with tobacco smoking, the dominant strand bias mutations on the non-coding strand (NTS) involves G-to-T, and to a lesser extent A-to-T, indicative of bulky adducts of guanine and adenine and which are consistent with reactive polyaromatic hydrocarbons (PAH) specifically (B[a]P adducts in cigarette smoke at G and A (Guttenplan et al 2012. Ewa and Danuta 2017). Thus, the strand bias here is explained by preferential transcription coupled repair (Hanawalt and Spivak 2008, TCR) of the transcribed strand (Denissenko et al 1996, 1998). The presence of exogenous adducts at G and A purines is consistent with enhanced TCR G-to-T strand biases at C>A trinucleotides and A-to-T strand biases at T>A trinucleotides in SBS5, SBS3 and SBS4.

It should be noted that there are also other types of endogenous and exogenous base modifications that are far less bulky than PAH/B[a]P adducts, such as ROS derived 8oxoG as well as O6-AlkG, O4-AltT (Swann 1990, Wirtz et al 2010, Malvessi et al 2017, Aitken et al 2020, Anderson et al 2024). If these alkylated bases are left unrepaired by BER or MMR, they are usually transcribed or replicated causing transition mutations G-to-A and T-to-C. In the case of the repair of 8oxoG lesions, causing G-to-T mutations, there is no formal transcriptional strand bias in their normal repair in mammalian cells *in vitro* (Thorslund et al 2002). Why then is the G-to-T mutation in excess of the C-to-A complement on the NTS in SBS18? Furthermore, it is known that uracil and abasic site lesions on transcribed template DNA strands can be copied over *in vitro* by RNA Pol II potentially causing G-to-A and other putative modifications such transversion G-to-C, G-to-T in the nascent transcript RNA (Kuraoka et al 2003).

Why are some C-site tri-nucleotide motifs more inclined to be G-to-A >>> C-to-T strand bias, yet at GCG, GCT, TCG there is more apparent C-to-T>>>G-to-A strand bias? This is typically apparent for example in SBS1 and lesser extent in SBS6. While these systematic strand biases in SBS1, SBS6 are more apparent than statistically significant it could be a case of easier access by the RNA Exosome (Basu et al 2011) allowing cytosine deamination on the TS at RNA:DNA hybrids? Is it a question of access by different cytosine deaminases to an unpaired cytosine on the TS or NTS? It may also relate to the relative strength of rC•dG and rG•dC base pairing in RNA:DNA hybrids within Transcription Bubbles (or R Loop)? and in certain 5' and 3' contexts (Huang et al 2009).

The origin of SBS19 poses interesting questions. It is not listed as "APOBEC" related, but it has a clear and pure transcriptional strand asymmetry signature (G-to-A >> C-to-T) in the trinucleotides of the C>T signature (at a number of motifs found in at least three cancers CNS-PiloAstro, Myeloid-MDS/MPN, Liver -HCC). If SBS2 and SBS13 are "APOBEC activity"-mediated why is SBS19 not listed as an AID/APOBEC signature? Indeed, this critical argument could be applied also to the molecular aetiology of SBS1 (not simply as 'spontaneous' deamination, but likely that the actively is driven in the vast majority of cases by AID/APOBEC deamination e.g. see Ito et al 2017). Active enzymatic deamination seems more likely given that the great majority of the breakage and reunion events of covalent bonds within a living cell are usually implemented in a regulated fashion by enzymatic catalysis. Is there any evidence for this assertion?

Finally, how to explain the clear flips or reversals in A>>T and G>>C strand biases, of varying magnitude, depending on the signature? (SBS17a, SBS17b, SBS21, SBS9).

These issues were at the forefront as the analyses unfolded.

Role of Sanger -Welcome Trust Institute.

It is acknowledged that The Sanger -Welcome Trust Institute at Hixton outside Cambridge has made big and important contributions to genomic medicine and cancer research in general. Their investment in next generation DNA sequencing, both whole genome and whole exome sequencing, has allowed the curation and assembly of the Catalogue of Somatic Mutations in Cancer (COSMIC). In 2013 Alexandrov et al reported the compendium of "Signatures of mutational processes in human cancer". They employed an algorithm or "mathematical" approach, a nonnegative matrix factorization (NMF) and 'model selection approach' (Berry et al., 2007). This method has extracted numerous 96 (x2) trinucleotide signatures in cancer exomes. Each trinucleotide sequence thus identifies 96 different contexts, the central C -site or T-site pyrimidine mutated base, but also the bases immediately 5' and 3'. This seems a perfectly reasonable approach. The latest online version for Single Base Substitution (SBS) Mutational Signatures (v3.4 October 2023) is at https://cancer.sanger.ac.uk/signatures/sbs/. Appended to each SBS signature is key information particularly on putative aetiology and likely molecular mechanism of formation where reasonably known. These are summarised and updated in Alexandrov et al 2020, and further in Otlu et al 2023.

In previous publications we have discussed how the endogenous origin of many cancer SBS signatures may be explained under an AID/APOBEC/ADAR deaminase mutation paradigm especially the dominant SBS5 signature. The aetiology for SBS5 is unknown, but it has also been variously described as a "Clock-like " cancer signature and also "flat'. SBS5 appears at significant strength (median mutations per Mb among tumours with the signature) in genomes of all cancer tissue types analysed. It displays a complex signature, across both A:T and G:C base pairs, and a clear dominance of mutations off pyrimidines C>T and T>C. It has been argued this broad A:T and G:C balanced signature is similar to, and consistent with, a dysregulated immunoglobulin (Ig) somatic hypermutation (SHM)-like mutation pattern (Steele and Lindley 2010, Lindley 2013, Lindley and Steele 2013, Lindley 2020, Steele, Franklin and Lindley 2024). Ig-SHM patterns are transcriptionally strand biased (Table 1A, Supplementary Figure S1) as is SBS5 (Table 1B) but SBS5 is less sharp and pronounced than Ig SHM strand bias. This type of strand bias is

symbolised by the generic symbols A>>T and G>>C (mutations of A exceed mutations of T, and mutations of G exceed mutations of C, when read from the non-transcribed or coding strand). This was first suggested in part in Steele and Lindley (2010) on the basis of the COSMIC data then available, and then extended and confirmed for many key strand biased patterns in TP53 sequence substrates (the 300 bp DNA binding region of TP53) in a range of human cancers (Lindley and Steele 2013). At that time the concept of Targeted Somatic Mutation (TSM) in codon-context was introduced which allowed a quite different analytical prognostic approach to cancer mutation signatures based on deaminase mutagenesis in transcribed protein coding regions (Lindley 2013). This in turn led to a detailed predictive (prognostic) analysis of the AID/APOBEC/ADAR driven TSM signatures in the TCGA database (The Cancer Genome Atlas) viz. on cancer progression associated signatures of high grade serous ovarian cancer (HGS-OvCa, Lindley et al 2016). More detailed theoretical considerations on diversification of deaminase binding domain specificities as a cancer matures and evolves can be found in Mamrot et al (2019). In addition, a machine learning approach and prediction of clinical outcomes employing more advanced cancer progression associated signatures is in Mamrot et al (2021). The TSM approach was reviewed in Steele (2016) and the TSM mechanism considered in the context of similar types of TSM signatures within 'passive passenger' Ig transgene sequences (rearranged VkOx1Jk5, expressed though inactive and non-antigen binding selected). This has all now been updated and reviewed in Steele, Franklin and Lindley 2024.

In Alexandrov et al (2013) 4,938,362 mutations were analysed from 7,042 human cancers and the NMF method extracted more than 20 distinct mutational SBS signatures. Many more cancer genomes have now been sequenced and this SBS list has since grown beyond 30 or more signatures (Alexandrov et al 2020, Otlu et al 2023 and Box 1), but the main original signatures published in 2013 are still evident or slightly subdivided. For the purposes of this paper many of the newer identified signatures are redundant and repetitive of major underlying mutational processes as the endogenous initiation events underlying many of the genome-wide cancer signatures appear driven largely by AID/APOBEC (cytosine-to-uracil, and to a much less extent, but very mutagenic, by cytosine-to-thymidine at meCpG) deamination sites, as well as ADAR1/2 (adenosine-to-inosine/hypoxanthine) deamination processes in RNA and DNA (Table 2,Table 3). For RNA mutations, we argue these are often mechanistically-coupled to cellular reverse transcriptase (RT)-mediated DNA repair by DNA polymerases eta (η) and putatively also by DNA polymerase theta (θ) (Franklin and Steele 2022, Steele Franklin and Lindley 2024). These mechanisms are expected to be usually executed on genomic DNA during post replication and transcriptional repair in a lesion site-directed fashion. Since DNA repair enzymes Polymerase-eta and Polymerase-theta are cellular reverse transcriptases as well as DNA dependent DNA polymerases, they can in theory execute target site reverse transcription (TSRT (cf. Luan et al 1993). They do this using the newly synthesised pre-mRNA as a copying template to repair the mutated patch in the genome at that site by synthesis of a cDNA of the transcribed strand (TS), and which can in theory, be integrated back into the genome (see update in Steele, Franklin and Lindley 2024). The putative Reverse Transcriptase Mechanism for SHM of Ig genes is shown in Supplementary Figure S1, and generalised for non-Ig loci in Figure 1a. This implies that at many target-site lesions repair synthesis may be mediated by a template switch by utilizing newly synthesised pre-mRNA at a given specific locus. Indeed, it has now been shown that human DNA Polymerase-eta can promote RNA-templated error-free repair of DNA double strand breaks (DSBs) in human cells (Chakraborty et al 2023), a function normally assigned to the BRCA1/2-associated Homologous Recombination (HR) DNA repair machinery. The final qualifying caveat is this: If the newly synthesised pre-mRNA now acting as a lesion repair copying template is itself carrying RNA base modifications, whether A-to-I (ADAR1/2) or C-to-U (APOBEC3A) or other unsuspected unrepaired endogenous base modifications (Reactive Oxygen Species, ROS) such as 8oxoG modifications , or others such as the common RNA uracil base isomeric modification pseudouridine (ψ), then any of these can potentially contribute to the mutation signature as assayed in the genomic DNA after reverse transcription. This focus on RNA intermediates acting as copying templates in the origin of genomic mutations may also help us understand the origin of specific SBS signatures that may occur in the vicinity of Replication Fork-R Loop conflicts (TRC) in cancer genomes (Bayona-Feliu et al 2023).

In addition to the cancer-wide dominance of SBS5, there are also specific SBS signatures at lower levels and incidence in most cancers (see figure 3 in Alexandrov et al 2020). The most often cited is the "Age Related" or "Clock-like Signature" SBS1 which highlights C-to-T mutations at CpG sites involving deamination of 5- methylcytosine (via spontaneous water deamination or by enzymatic deamination). This appears at a lower and variable level in most if not all cancer types. However, the most cited signatures and labelled as the "APOBEC" signatures (SBS2, SBS13) appears in a significant proportion of all cancer types (approx. 72 %).

A number of issues expanded on from the main text are now dealt with in some detail.

A. Lagging and Leading Strands of the Replication Forks

Replication forks (RF) are frequently stalled at sites of DNA damage, and in rapidly dividing cancer cells RFs are under frequent replicative stress. At the COSMIC website each SBS signatures is also assessed for any effects on 'Replicational strand asymmetry'. This would be particularly likely at Transcription Replication Fork conflicts (TRC, Bayona-Feliu et al 2023). Lagging and leading Strands of the RF are a potential rich source of C-site deamination at ssDNA substrates mainly on the lagging strands (Chan et al 2015, Seplyarskiy et al 2016, Haradhvala et al 2016, Mertz et al 2017, Buisson et al 2017). They allow transient deaminase attack on exposed unpaired C-sites in the ssDNA regions and in the unpaired loops of transient forming DNA stem loops, not only for putative off target AID deamination (Senigl et al 2017) but also for APOBEC3A deamination (YT<u>C</u>W), APOBEC3B deaminations (RT<u>C</u>) and single stranded regions can be deaminated by APOBEC3H (Chan et al 2015, Bruisson et al 2017, Sanchez et al 2024). This is confirmed by the strand topography analysis in Otlu et al (2023) who are explicit concerning the likely origins of extracted SBS2 and SBS13 signatures, attributed to aberrant APOBEC3 family deaminations. SBS2/SBS13 are enriched on the lagging strand in all cancer types, and these signatures display little transcriptional strand asymmetry (which goes against the larger trend seen in the dominant global SBS5 transcriptional strand bias signature).

A key issue however is whether AID/APOBEC-deaminase substrates at C-sites in RF lagging strands contribute significantly to the many observed transcription strand biased signals observed in the SBS profiles evaluated here. This is because even if there are 'local' RF strand bias effects in different potential off-target Ig SHM-like sites across the genome (Senigl et al 2017), such signals would be balanced out across the genome. The many origins of replication in mammalian genomic duplication and the known bi-directional replication ensures such mutation signals do not contribute to the transcriptional strand asymmetries analysed here, apart from the fact they may blunt or diminish the extent of the transcriptional strand bias. Such bi-directional replication processes make it difficult to envisage how a consistent transcriptional strand biased mutation pattern could emerge across the transcribed protein coding (genic) regions of cancer genomes. This is confirmed by an inspection of the replication strand asymmetry signals across many prominent SBS signatures. For example, the set of C>T trinucleotides in SBS19 has a prominent transcriptional strand biased pattern, yet no replication strand bias on the lagging strand at RFs. Similarly, SBS12 displays strong A-to-G over T-to-C transcriptional strand bias (at the set of T>C trinucleotides) but no replication strand bias signal. It is for this reason that most of the interesting transcriptional strand biased mutations at A:T base pairs most likely arise at R Loops, probably often in the context of TRC (Bayona-Feliu et al 2023). Replication plays a key role in replicating simple base mis-pairs in DNA preserving the observed single base substitutions, and thus generating the transcriptional strand biased SBS profiles.

B. Other features of the SBS5 signature in selected cancers which deserve comment and informed speculation (Table 2).

ColoRect-AdenoCA: The very high T-to-C proportion in the mutation spectrum (26.8 % of all mutations) exceeds the percent A-to-G mutations (26.3%). A possible reason is that in rapidly progressing cancers there is a large number of putative R Loops that have been collapsed (loss nascent pre-mRNA). In rapidly dividing cells this might suggest that annealed RNA:DNA hybrids in the R Loop that have been DNA deaminated to Inosine (Hypoxanthine) - and given the loss of the pre-mRNA- the only the A-

to-I product in DNA could be unrepaired Hypoxanthine. These are rapidly replicated unrepaired by the *POLE/POLD* replication apparatus generating excess numbers of T-to-C mutations on the NTS. This explanation fits the observed data.

Eso-AdenoCA: The relatively high proportion G-to-T mutations (8%) suggests TCR clearing bulky smoke adducts from transcribed strand is the cause (and for high G-to-T and C-to-A in total).

Liver-HCC: This has always been extremely interesting. Elsewhere we have argued that this is not a vague and unknown "Transcription Couple Damage" (TCD) of Adenines on the NTS (Haradhvala et al 2016), but the likely direct manifestation of the ADAR1 deamination process on nascent pre-mRNA in ADAR1 High expressed HCC tumours. These are then rendered as DNA mutations via the coupled TSRT process (Lindley and Steele 2020). All the other features of global A>>T and G>>C strand bias are clearly operating (Table 2).

Lung-SCC: There are two features that appear to buck the trends. The first is the high A-to-C mutations and loss of T-to-G > A-to-C strand bias. It is possible that in these tumours bulky adducts of adenine (smoking) are in the extreme and the strand bias is now conventional TCR clearance from transcribed strand as discussed. The second are the very high mutations of G-to-C and C-to-G (12%). REV1 is the primary Y family translesion enzyme repairing such damage (reviewed in Steele 2009). A plausible explanation would thus include defects in REV1 itself in such tumours, or aberrant functional effects associated with REV1 activity.

Lymph-BNHL: The very high T-to-C (15%) suggests a similar explanation as for Coloctrect-AdenoCA. In these progressing cancers there is a likely large number of putative R Loops that on ADAR-mediated A-to-I facilitated collapse (and thus loss of nascent pre-mRNA), the unrepaired Hypoxanthine on the TS are replicated unrepaired generating excess numbers of T-to-C mutations on the NTS. This explanation fits the observed data.

Lymph-CLL: As with Lung-SCC there is a high level of G-to-C and C-to-G mutations (10.7%). Thus a plausible explanation would thus include defects in REV1 or functional defects associated with REV1 activity.

Panc-AdenoCA : The relatively high A-to-C suggest a conventional TCR clearance process of bulky adducts of adenine on the TS.

Skin- Melanoma: Why the high level of A-to-C and T-to-G is unknown. One suggestion might be that in UV exposure-associated skin cancer postulated pseudourinylation (ψ) is far higher than in normal cancers?

Stomach-AdenoCA: There are very high T-to-C, T-to-A, A-to-C and T-to-G mutations that exceed the trends in other tumours. A combination of explanations involving ADAR1/2 deaminations at R Loops and excessive pseudourinylation (ψ)?

Uterus-AdenoCA: A plausible explanation for higher than expected T-to-A level is not easily advanced, but may be found in Hypoxanthine Wobble Base pairing in DNA replication producing the NTS at ADAR facilitated collapsed R Loops.

Section 3: Supplementary tables

Supplementary TableS1 SBS5 by cancer tissue type presented as "Types of Mutation" tables

In Strand Bias Summary main text Table 2 +++ means p<0.001,++ P<0.01,+ p<0.05, NS p>0.05.

Flip means Reverse Direction Strand Bias to dominant global strand bias.

N is Total number somatic mutations in that cancer type

https://cancer.sanger.ac.uk/signatures/sbs/sbs5/#transcriptional-strand-asymmetry

There must be at least 1000 mutations on the strands.

Signature and mutation type must have at least 5% of all signature mutations on the strands.

Odds ratio between fold change of real mutations and fold change of simulated mutations must be at least 1.1.

Highlighted is Unexpected Flip or Reverse Strand Bias or NS

**SBS5**	**As read on Coding or Non Transcribed Strand**							
**All**		**To**						
	**From**	**A**	**T**	**C**	**G**	**Total**		
	**A**		108	86	274	**468**	A>>T NS	A-to-G > T-to-C NS
	**T**	118		277	104	**499**		T-to-G > A-to-C NS
	**C**	95	198		45	**338**	G>>C NS	G-to-A > C-to-T NS
	**G**	187	116	52		**355**		G-to-T > C-to-A NS
						**1660**		
**SBS5**	**As read on Coding or Non Transcribed Strand**							
**Biliary-AdenoCA**		**To**						
	**From**	**A**	**T**	**C**	**G**	**Total**		
	**A**		4585	2624	12541	**19750**	A>>T 1.1x p<0.001	A-to-G > T-to-C 1.2x p<0.001
	**T**	4444		10865	3092	**18401**		T-to-G >A-to-C 1.2x p<0.01
	**C**	3318	9337		2203	**14858**	G>>C 1.1x p<0.001	G-to-A > C-to-T 1.1x p<0.05
	**G**	9797	3661	2270		**15728**		G-to-T > C-to-A 1.1x p<0.01
						**68737**		C-to-G > G-to-C 0.97x NS
**SBS5**	**As read on Coding or Non Transcribed Strand**							
**Bladder-TCC**		**To**						
	**From**	**A**	**T**	**C**	**G**	**Total**		
	**A**		3693	2749	12570	**19012**	A>>T 1.2x p<0.001	A-to-G > T-to-C 1.3x p<0.001
	**T**	3288		9688	3105	**16081**		T-to-G >A-to-C 1.1x p <0.01
	**C**	3787	10768		3296	**17851**	G>>C 1.1x p<0.001	G-to-A > C-to-T 1.1x p<0.001
	**G**	11645	4639	3540		**19824**		G-to-T > C-to-A 1.2x p<0.001
						**72768**		C-to-G > G-to-C 0.93x NS
**SBS5**	**As read on Coding or Non Transcribed Strand**							
**Bone-Osteosarc**		**To**						
	**From**	**A**	**T**	**C**	**G**	**Total**		
	**A**		539	233	1755	**2527**	A>>T 1.2x p<0.001	A-to-G > T-to-C 1.2x p<0.001
	**T**	472		1472	239	**2183**		T-to-G > A-to-C NS
	**C**	157	1049		343	**1549**	G>>C 1.1x p <0.05	G-to-A > C-to-T 1.2x p<0.05
	**G**	1208	239	364		**1811**		G-to-T > C-to-A 1.5x p<0.01
						**8070**		C-to-G > G-to-C 0.94x NS
**SBS5**	**As read on Coding or Non Transcribed Strand**							
**Bone-Benign**		**To**						
	**From**	**A**	**T**	**C**	**G**	**Total**		
	**A**		59	125	262	**446**	A>>T NS	A-to-G > T-to-C NS
	**T**	50		227	119	**396**		T-to-G > A-to-C NS
	**C**	58	340		105	**503**	G>>C NS	G-to-A > C-to-T NS
	**G**	357	82	86		**525**		G-to-T > C-to-A NS
						**1870**		C-to-G > G-to-C 1.2x NS

SBS5	As read on Coding or Non Transcribed Strand							
**Breast-Cancer**		**To**						
	**From**	**A**	**T**	**C**	**G**	**Total**		
	**A**		24085	17260	65011	**106356**	A>>T 1.1x p<0.001	A-to-G > T-to-C 1.2x p<0.001
	**T**	23185		55646	20857	**99688**		T-to-G > A-to-C 1.2x p<0.001
	**C**	18029	55268		17138	**90435**	G>>C 1.1x p<0.001	G-to-A > C-to-T 1.04x p<0.001
	**G**	57256	22364	16091		**95711**		G-to-T > C-to-A 1.2x p<0.001
						**392190**		C-to-G > G-to-C 1.1x p<0.001

SBS5	As read on Coding or Non Transcribed Strand							
**Cervix-Cancer**		**To**						
	**From**	**A**	**T**	**C**	**G**	**Total**		
	**A**		870	444	1748	**3062**	A>>T NS	A-to-G > T-to-C NS
	**T**	840		1643	570	**3053**		T-to-G > A-to-C 1.3x p<0.01
	**C**	885	1926		523	**3334**	G>>C 1.1x p<0.05	G-to-A > C-to-T NS
	**G**	2002	1011	583		**3596**		G-to-T > C-to-A 1.2x p<0.01
						**13045**		C-to-G > G-to-C 0.9x NS

SBS5	As read on Coding or Non Transcribed Strand							
**CNS-GBM**		**To**						
	**From**	**A**	**T**	**C**	**G**	**Total**		
	**A**		1715	879	5379	**7973**	A>>T 1.1x p<0.01	A-to-G > T-to-C 1.1x P<0.001
	**T**	1691		4788	1026	**7505**		T-to-G > A-to-C 1.2x p<0.05
	**C**	1766	3445		1475	**6686**	G>>C 1.2x p<0.001	G-to-A > C-to-T 1.2x p<0.001
	**G**	4118	2137	1458		**7713**		G-to-T > C-to-A 1.2x p<0.001
						**29877**		C-to-G > G-to-C 1x NS

SBS5	As read on Coding or Non Transcribed Strand							
**CNS-Medullo**		**To**						
	**From**	**A**	**T**	**C**	**G**	**Total**		
	**A**		1505	919	5462	**7886**	A>>T 1.1 p<0.05	A-to-G > T-to-C 1.1x p<0.001
	**T**	1474		4964	1087	**7525**		T-to-G > A-to-C 1.2x p<0.01
	**C**	1038	4113		1055	**6206**	G>>C 1.2x p<0.001	G-to-A > C-to-T 1.1x p<0.001
	**G**	4579	1273	1338		**7190**		G-to-T > C-to-A 1.2x p<0.001
						**28807**		C-to-G < G-to-C 1.3x p<0.001

SBS5	As read on Coding or Non Transcribed Strand							
**CNS-Oligo**		**To**						
	**From**	**A**	**T**	**C**	**G**	**Total**		
	**A**		470	378	2336	**3184**	A>>T 1.3x p<0.001	A-to-G > T-to-C 1.6x p<0.001
	**T**	406		1511	528	**2445**		T-to-G > A-to-C 1.4x p<0.001
	**C**	475	1553		596	**2624**	G>>C 1.2x p<0.001	G-to-A > C-to-T 1.3x p<0.05
	**G**	1960	593	454		**3007**		G-to-T > C-to-A 1.3x p<0.05
						**11260**		C-to-G > G-to-C 1.3x p<0.01

SBS5	As read on Coding or Non Transcribed Strand							
**CNS-PiloAstro**		**To**						
	**From**	**A**	**T**	**C**	**G**	**Total**		
	**A**		197	81	598	**876**	A>>T 1.2x p<0.05	A-to-G > T-to-C 1.2x 0<0.5
	**T**	142		495	113	**750**		T-to-G > A-to-C NS
	**C**	168	370		132	**670**	G>>C NS	G-to-A > C-to-T NS
	**G**	421	195	101		**717**		G-to-T > C-to-A NS
						**3013**		C-to-G > G-to-C NS

SBS5	As read on Coding or Non Transcribed Strand							
ColoRect-AdenoCA		To						
	From	A	T	C	G	Total		
	A		10842	3492	81383	95717	A<<T 1.015x p<0.001	A-to-G<<T-to-C 1.017x p<0.05
	T	10438		82771	3926	97135		T-to-G > A-to-C 1.2x p<0.001
	C	9443	39556		6238	55237	G>>C 1.1x p<0.001	G-to-A > C-to-T 1.1x p<0.001
	G	44739	10325	6207		61271		G-to-T > C-to-A 1.1x p<0.001
						309360		C-to-G > G-to-C NS

SBS5	As read on Coding or Non Transcribed Strand							
ESCC		To						
	From	A	T	C	G	Total		
	A		40545	29804	111942	182291	A>>T 1.1x p<0.001	A-to-G > T-to-C 1.2x p<0.001
	T	36909		93514	32784	163207		T-to-G > A-to-C 1.1x p<0.001
	C	19194	80212		30176	129582	G>>C 1.06x p<0.001	G-to-A > C-to-T 1.04x p<0.001
	G	83324	22889	31279		137492		G-to-T > C-to-A 1.2x p<0.001
						612572		C-to-G < G-to-C 1.04x p<0.01

SBS5	As read on Coding or Non Transcribed Strand							
Eso-AdenoCA		To						
	From	A	T	C	G	Total		
	A		11201	4794	25536	41531	A>>T NS	A-to-G > T-to-C NS
	T	11751		25120	5209	42080		T-to-G > A-to-C 1.1x p<0.01
	C	13315	27355		10270	50940	G>>C 1.03x p<0.001	G-to-A > C-to-T NS
	G	27616	14819	10205		52640		G-to-T > C-to-A 1.1x p<0.001
						187191		C-to-G > G-to-C NS

SBS5	As read on Coding or Non Transcribed Strand							
Head-SCC		To						
	From	A	T	C	G	Total		
	A		4321	2980	14508	21809	A>>T 1.3x p<0.001	A-to-G > T-to-C 1.4x p<0.001
	T	3449		10508	3142	17099		T-to-G > A-to-C 1.1x NS
	C	2003	11444		2972	16419	G>>C 1.1x p<0.001	G-to-A > C-to-T 1.1x p<0.001
	G	12201	2419	3390		18010		G-to-T > C-to-A 1.2x p<0.001
						73337		C-to-G < G-to-C 1.2x p<0.001

SBS5	As read on Coding or Non Transcribed Strand							
Kidney-RCC		To						
	From	A	T	C	G	Total		
	A		1016	511	5279	6806	A>>T NS	A-to-G > T-to-C 1.1x p<0.05
	T	1217		4975	500	6692		T-to-G > A-to-C NS
	C	1124	3036		662	4822	G>>C 1.1x p<0.001	G-to-A > C-to-T 1.1x p<0.05
	G	3260	1314	740		5314		G-to-T > C-to-A 1.2x p<0.01
						23634		C-to-G > G-to-C 0.9x NS

SBS5	As read on Coding or Non Transcribed Strand							
Liver-HCC		To						
	From	A	T	C	G	Total		
	A		77573	46403	210192	334168	A>>T 1.4x p<0.001	A-to-G > T-to-C 1.7x p<0.001
	T	65273		124000	53223	242496		T-to-G > A-to-C 1.2x p<0.001
	C	43679	159407		54595	257681	G>>C 1.1x p<0.001	G-to-A > C-to-T 1.1x p<0.001
	G	172167	55144	48006		275317		G-to-T > C-to-A 1.3x p<0.001
						1109662		C-to-G > G-to-C 1.1x p<0.001

SBS5	As read on Coding or Non Transcribed Strand							
Lung-AdenoCA		To						
	From	A	T	C	G	Total		
	A		2925	10279	41852	55056	A>>T 1.3x p<0.001	A-to-G > T-to-C 1.4x p<0.001
	T	2242		31003	10524	43769		T-to-G > A-to-C 1.02x p<0.001
	C	1239	32405		6648	40292	G>>C 1.03x p<0.01	G-to-A > C-to-T NS
	G	32978	1502	7139		41619		G-to-T > C-to-A 1.2x p<0.001
						180736		C-to-G < G-to-C 1.1x p<0.01

**SBS5**	**As read on Coding or Non Transcribed Strand**							
**Lung-SCC**		**To**						
	**From**	**A**	**T**	**C**	**G**	**Total**		
	**A**		9265	13992	57262	**80519**	A>>T 1.3x p<0.001	A-to-G > T-to-C 1.4x p<0.001
	**T**	7335		42076	13363	**62774**		T-toG < A-to-C 1.05x P<0.01
	**C**	900	49553		15483	**65936**	G>>C 1.1x p<0.001	G-to-A > C-to-T 1.1x p<0.001
	**G**	51253	1188	17776		**70217**		G-to-T > C-to-A 1.3x p<0.001
						**279446**		G-to-C,C-to-G muts very high!
								C-to-G < G-to-GC 1.2x p<0.001

**SBS5**	**As read on Coding or Non Transcribed Strand**							
**Lymph-BNHL**		**To**						
	**From**	**A**	**T**	**C**	**G**	**Total**		
	**A**		3368	2423	12882	**18673**	A>>T NS	A-to-G > T-to-C 0.99x NS
	**T**	3402		12996	2649	**19047**		T-to-G > A-to-C 1.1x p<0.05
	**C**	3489	15737		4699	**23925**	G>>C 1.05x p<0.001	G-to-A > C-to-T 1.04x p<0.01
	**G**	16419	3889	4581		**24889**		G-to-T > C-to-A 1.1x p≤0.001
						**86534**		C-to-G > G-to-C NS

**SBS5**	**As read on Coding or Non Transcribed Strand**							
**Lymph-CLL**		**To**						
	**From**	**A**	**T**	**C**	**G**	**Total**		
	**A**		2075	2122	12156	**16353**	A>>T 1.1x p<0.001	A-to-G > T-to-C 1.2x p<0.001
	**T**	1672		10544	2521	**14737**		T-to-G > A-to-C 1.2x p<0.001
	**C**	2176	12689		3823	**18688**	G>>C 1.03x p<0.05	G-to-A > C-to-T 1.1x p<0.01
	**G**	13347	2364	3536		**19247**		G-to-T > C-to-A 1.1x p≤0.05
						**69025**		C-to-G > G-to-C 1.1x p<0.05

**SBS5**	**As read on Coding or Non Transcribed Strand**							
**Myeloid-AML**		**To**						
	**From**	**A**	**T**	**C**	**G**	**Total**		
	**A**		250	153	739	**1142**	A>>T NS	A-to-G > T-to-C 1.3x p<0.01
	**T**	247		579	197	**1023**		T-to-G > A-to-C NS
	**C**	230	834		241	**1305**	G>>C NS	G-to-A > C-to-T NS
	**G**	915	256	185		**1356**		G-to-T > C-to-A NS
						**4826**		C-to-G > G-to-C NS

**SBS5**	**As read on Coding or Non Transcribed Strand**							
**Myeloid-MPN**		**To**						
	**From**	**A**	**T**	**C**	**G**	**Total**		
	**A**		1045	731	2506	**4282**	A>>T 1.1x p<0.01	A-to-G > T-to-C 1.4x p<0.001
	**T**	1074		1855	947	**3876**		T-to-G > A-to-C 1.3x p<0.001
	**C**	1282	2842		839	**4963**	G>>C 1.2x p<0.001	G-to-A > C-to-T 1.2x p<0.001
	**G**	3371	1511	807		**5689**		G-to-T > C-to-A 1.2x p<0.01
						**18810**		C-to-G > G-to-C NS

**SBS5**	**As read on Coding or Non Transcribed Strand**							
**Ovary-AdenoCA**		**To**						
	**From**	**A**	**T**	**C**	**G**	**Total**		
	**A**		169	88	2166	**2423**	A>>T 1.3x p<0.001	A-to-G > T-to-C 1.4x p<0.001
	**T**	202		1571	105	**1878**		T-to-G > A-to-C NS
	**C**	490	1822		577	**2889**	G>>C NS	G-to-A > C-to-T NS
	**G**	1770	531	596		**2897**		G-to-T > C-to-A NS
						**10087**		C-to-G > G-to-C NS

**SBS5**	**As read on Coding or Non Transcribed Strand**							
**Panc-AdenoCA**		**To**						
	**From**	**A**	**T**	**C**	**G**	**Total**		
	**A**		11060	7171	31994	**50225**	A>>T 1.1x p<0.001	A-to-G > T-to-C 1.1x p<0.001
	**T**	10982		28218	8610	**47810**		T-to-G > A-to-C 1.03x p<0.05
	**C**	5928	27254		6544	**39726**	G>>C 1.04x p<0.001	G-to-A > C-to-T 1.03x p<0.05
	**G**	27963	7270	6226		**41459**		G-to-T > C-to-A 1.2x p<0.001
						**179220**		C-to-G > G-to-C 1.5x p<0.05

SBS5	As read on Coding or Non Transcribed Strand							
Prost-AdenoCA		To						
	From	A	T	C	G	Total		
	A		8006	5430	26346	39782	A>>T 1.1x p<0.001	A-to-G > T-to-C 1.3x p<0.001
	T	7901		20902	6762	35565		T-to-G > A-to-C 1.3x p<0.001
	C	6977	18257		6051	31285	G>>C 1.1x p<0.001	G-to-A > C-to-T NS
	G	18641	8773	5291		32705		G-to-T > C-to-A 1.3x p<0.001
						139337		C-to-G > G-to-C 1.1x p<0.001

SBS5	As read on Coding or Non Transcribed Strand							
Skin-Melanoma		To						
	From	A	T	C	G	Total		
	A		7788	13595	53212	74595	A<<T 1.02x p<0.01	A-to-G > T-to-C 1.04x p<0.001
	T	7541		51000	17807	76348		T-to-G > A-to-C 1.3x p<0.001
	C	19415	25492		23566	68473	G>>C 1.4x p<0.001	G-to-A > C-to-T 1.04x p<0.01
	G	26506	21655	23192		71353		G-to-T > C-to-A 1.1x p<0.001
						290769		C-to-G > G-to-C NS

SBS5	As read on Coding or Non Transcribed Strand							
SoftTissue-Liposarc		To						
	From	A	T	C	G	Total		
	A		163	135	531	829	1.2x p<0.05	A-to-G > T-to-C 1.2x p<0.05
	T	151		427	142	720		T-to-G > A-to-C NS
	C	16	633		136	785	G>>C NS	G-to-A > C-to-T NS
	G	606	20	128		754		G-to-T > C-to-A NS
						3088		C-to-G > G-to-C NS

SBS5	As read on Coding or Non Transcribed Strand							
Stomach-AdenoCA		To						
	From	A	T	C	G	Total		
	A		25284	12400	51999	89683	A>>T NS	A-to-G > T-to-C 1.04x p<0.001
	T	25443		49851	13903	89197		T-to-G > A-to-C 1.1x p<0.001
	C	16256	55688		14273	86217	G>>C 1.03x p<0.001	G-to-A > C-to-T 1.02x p<0.05
	G	56777	17863	14043		88683		G-to-T > C-to-A 1.1x p<0.001
						353780		C-to-G > G-to-C NS

SBS5	As read on Coding or Non Transcribed Strand							
Thy-AdenoCA		To						
	From	A	T	C	G	Total		
	A		776	189	2542	3507	A>>T 1.5x p<0.001	A-to-G > T-to-C 1.6x p<0.001
	T	513		1591	271	2375		T-to-G > A-to-C 1.4x p<0.01
	C	506	2033		448	2987	G<<C 1.08x p<0.05	G-to-A < C-to-T 1.08x NS
	G	1881	506	367		2754		G-to-T > C-to-A NS
						11623		C-to-G > G-to-C 1.2x p<0.05

SBS5	As read on Coding or Non Transcribed Strand							
Uterus-AdenoCA		To						
	From	A	T	C	G	Total		
	A		5819	2299	4172	12290	A<<T 1.1x p<0.001	A-to-G > T-to-C 1.8x p<0.05
	T	7093		3876	2637	13606		T-to-G > A-to-C 1.15x p<0.001
	C	2922	6302		2005	11229	G>>C 1.5x p<0.01	G-to-A > C-to-T NS
	G	6560	3155	2081		11796		G-to-T > C-to-A 1.08x p<0.05
						48921		C-to-G > G-to-C NS

Supplementary Table S2 SBS3 by cancer tissue type prsented as "Types of Mutation" tables

https://cancer.sanger.ac.uk/signatures/sbs/sbs3/#transcriptional-strand-asymmetry

There must be at least 1000 mutations on the strands.

Signature and mutation type must have at least 5% of all signature mutations on the strands.

Odds ratio between fold change of real mutations and fold change of simulated mutations must be at least 1.1.

Highlighted is Unexpected Flip or Reverse Strand Bias or NS

Highlight Specific signature, "Smoking signature" code for TCR repair bulky adducts

SBS3	As read on Coding or Non Transcribed Strand							
Bone-Osteosarc		To						
	From	A	T	C	G	Total		
	A		423	94	57	574	A>>T NS	A-to-G > T-to-C NS
	T	381		46	99	526		T-to-G > A-to-C NS
	C	178	0		298	476	G>>C NS	G-to-A > C-to-T 0 mutations
	G	0	186	308		494		G-to-T > C-to-A NS
						2070		C-to-G > G-to-C NS

SBS3	As read on Coding or Non Transcribed Strand							
Breast-Cancer		To						
	From	A	T	C	G	Total		
	A		37036	24122	37688	98846	A>>T NS	A-to-G > T-to-C 1.03x p<0.01
	T	35957		36427	26243	98627		T-to-G > A-to-C 1.01x p<0.001
	C	40276	31666		40583	112525	G>>C 1.1x p<0.001	G-to-A > C-to-T 1.06x p<0.001
	G	33520	47784	42035		123339		G-to-T > C-to-A 1.2x p<0.001
						433337		C-to-G < G-to-C 1.04x p<0.001

SBS3	As read on Coding or Non Transcribed Strand							
CNS-Medullo		To						
	From	A	T	C	G	Total		
	A		237	105	266	608	A>>T NS	A-to-G > T-to-C NS
	T	245		275	112	632		T-to-G > A-to-C NS
	C	239	144		172	555	G>>C 1.3x p<0.01	G-to-A > C-to-T NS
	G	159	283	256		698		G-to-T > C-to-A NS
						2493		C-to-G < G-to-C 1.5x p<0.01

SBS3	As read on Coding or Non Transcribed Strand							
ESCC		To						
Smoking signature?	From	A	T	C	G	Total		A-to-T>T-to-A 1.07x p<0.001
	A		18654	9321	8390	36365	A>>T 1.02x NS	A-to-G > T-to-C NS
	T	17478		8200	10053	35731		T-to-G > A-to-C 1.08x p<0.001
	C	13140	5246		18588	36974	G>>C 1.1x p<0.001	G-to-A > C-to-T 1.07x p<0.05
	G	5605	15215	20128		40948		G-to-T > C-to-A 1.2x p<0.001
						150018		C-to-G < G-to-C 1.08x p<0.001
								C-to-G, G-to-C both very high

SBS3	As read on Coding or Non Transcribed Strand							
Eso-AdenoCA		To						
Smoking signature?	From	A	T	C	G	Total		A-to-T>T-to-A NS
	A		917	326	17	1260	A<<T NS	A-to-G > T-to-C NS
	T	972		12	360	1344		T-to-G > A-to-C NS
	C	1892	37		1647	3576	G>>C 1.09x p<0.01	G-to-A > C-to-T NS
	G	42	2249	1613		3904		G-to-T > C-to-A 1.2x <0.001
						10084		C-to-G > G-to-C NS

SBS3	As read on Coding or Non Transcribed Strand							
Head-SCC		To						
Smoking signature?	From	A	T	C	G	Total		A-to-T>T-to-A 1.2x p<0.05
	A		943	361	214	1518	A>>T 1.1x p<0.05	A-to-G > T-to-C NS
	T	805		168	389	1362		T-to-G > A-to-C NS
	C	903	281		522	1706	G>>C 1.1x p<0.01	G-to-A > C-to-T NS
	G	346	1130	473		1949		G-to-T > C-to-A 1.3x p<0.001
						6535		C-to-G > G-to-C NS

**SBS3**	**As read on Coding or Non Transcribed Strand**							
**Liver-HCC**		**To**						
Smoking signature?	**From**	**A**	**T**	**C**	**G**	**Total**		A-to-T>T-to-A NS
	**A**		375	24	0	**399**	A>>T NS	A-to-G > T-to-C NS
	**T**	329		0	20	**349**		T-to-G > A-to-C NS
	**C**	155	0		207	**362**	G>>C 1.3x p<0.01	G-to-A > C-to-T NS
	**G**	0	241	234		**475**		G-to-T > C-to-A 1.6x p<0.01
						**1585**		C-to-G > G-to-C NS

**SBS3**	**As read on Coding or Non Transcribed Strand**							
**Lung-AdenoCA**		**To**						
	**From**	**A**	**T**	**C**	**G**	**Total**		
	**A**		275	196	182	**653**	A>>T NS	A-to-G > T-to-C NS
	**T**	274		196	189	**659**		T-to-G > A-to-C NS
	**C**	328	180		161	**669**	G>>C NS	G-to-A > C-to-T NS
	**G**	185	376	188		**749**		G-to-T > C-to-A NS
						**2730**		C-to-G > G-to-C NS

**SBS3**	**As read on Coding or Non Transcribed Strand**							
**Ovary-AdenoCA**		**To**						
Smoking signature?	**From**	**A**	**T**	**C**	**G**	**Total**		A-to-T>T-to-A 1.1x p<0.001
	**A**		16109	7847	10713	**34669**	A>>T 1.04x p<0.001	A-to-G > T-to-C 1.06x p<0.01
	**T**	14706		10086	8466	**33258**		T-to-G > A-to-C 1.08x p<0.01
	**C**	14499	8023		18754	**41276**	G>>C 1.1x p<0.001	G-to-A > C-to-T 1.1x p<0.001
	**G**	8862	17569	19259		**45690**		G-to-T > C-to-A 1.2x p<0.001
						**154893**		C-to-G < G-to-C 1.03x NS
								C-to-G, G-to-C both very high

**SBS3**	**As read on Coding or Non Transcribed Strand**							
**Panc-AdenoCA**		**To**						
Smoking signature?	**From**	**A**	**T**	**C**	**G**	**Total**		A-to-T>T-to-A 1.1x p <0.001
	**A**		5886	2053	1150	**9089**	A>>T 1.06x p<0.01	A-to-G > T-to-C NS
	**T**	5366		1022	2209	**8597**		T-to-G > A-to-C NS
	**C**	3705	1055		4996	**9756**	G>>C 1.08x p<0.001	G-to-A > C-to-T NS
	**G**	1152	4146	5195		**10493**		G-to-T > C-to-A 1.1x p<0.001
						**37935**		C-to-G > G-to-C

**SBS3**	**As read on Coding or Non Transcribed Strand**							
**Panc-Endocrine**		**To**						
Smoking signature?	**From**	**A**	**T**	**C**	**G**	**Total**		A-to-T>T-to-A NS
	**A**		488	118	44	**650**	A>>T NS	A-to-G > T-to-C NS
	**T**	495		43	148	**686**		T-to-G > A-to-C NS
	**C**	267	0		439	**706**	G>>C NS	G-to-A > C-to-T NS
	**G**	0	386	421		**807**		G-to-T > C-to-A 1.4x p≤0.001
						**2849**		C-to-G > G-to-C NS

**SBS3**	**As read on Coding or Non Transcribed Strand**							
**Prost-AdenoCA**		**To**						
	**From**	**A**	**T**	**C**	**G**	**Total**		A-to-T>T-to-A NS
	**A**		661	39	14	**714**	A>>T NS	A-to-G > T-to-C NS
	**T**	608		10	55	**673**		T-to-G > A-to-C NS
	**C**	43	1		224	**268**	G>>C 1.3x p≤0.01	G-to-A > C-to-T NS
	**G**	3	60	296		**359**		G-to-T > C-to-A NS
						**2014**		C-to-G < G-to-C 1.3x p<0.05

**SBS3**	**As read on Coding or Non Transcribed Strand**							
**Stomach-AdenoCA**		**To**						
Smoking signature?	**From**	**A**	**T**	**C**	**G**	**Total**		A-to-T>T-to-A NS
	**A**		5835	2815	2317	**10967**	A>>T NS	A-to-G > T-to-C NS
	**T**	5728		2256	3088	**11072**		T-to-G > A-to-C 1.1x p<0.05
	**C**	4475	2284		6760	**13519**	G>>C 1.08x p<0.001	G-to-A > C-to-T NS
	**G**	2363	5150	7170		**14683**		G-to-T > C-to-A 1.2x p<0.001
						**50241**		C-to-G < G-to-C 1.06x p<0.05
								C-to-G, G-to-C both very high

**SBS3**	**As read on Coding or Non Transcribed Strand**							
**Uterus-AdenoCA**		**To**						
	**From**	**A**	**T**	**C**	**G**	**Total**		A-to-T>T-to-A NS
	**A**		269	232	413	**914**	A>>T NS	A-to-G > T-to-C NS
	**T**	262		374	234	**870**		T-to-G > A-to-C NS
	**C**	323	267		337	**927**	G>>C NS	G-to-A > C-to-T NS
	**G**	225	386	341		**952**		G-to-T > C-to-A NS
						**3663**		C-to-G < G-to-C NS

Supplementary Table S3 Strand Bias Summary SBS7 -Skin Melanoma

Transcriptional Strand Asymmetries harvested from

https://cancer.sanger.ac.uk/signatures/sbs/

There must be at least 1000 mutations on the strands.

Signature and mutation type must have at least 5% of all signature mutations on the strands.

Odds ratio between fold change of real mutations and fold change of simulated mutations must be at least 1.1.

In SBS7a 99% of 7,010,879 mutations are C-to-T, G-to-A

In SBS7b 72% of 20167 mutations are C-to-T, G-to-A

In SBS7c 96% of 252,744 mutations are at A:T base pairs

In SBS7d 98% of 133,806 mutations are at A:T base pairs

**SBS7a**	**As read on Coding or Non Transcribed Strand**							
		**To**						
	**From**	**A**	**T**	**C**	**G**	**Total**		A-to-T > T-to-A 1.1x p<0.001
	**A**		19605	3057	5957	**28619**	A>>T NS	A-to-G < T-to-C NS
	**T**	17635		6502	3931	**28068**		T-to-G >A-to-C 1.3x p<0.001
	**C**	3833	3997003		664	**4001500**	G<<C 1.4x p<0.001	G-to-A < C-to-T 1.4x p<0.001
	**G**	2948026	3957	709		**2952692**		G-to-T > C-to-A NS
						**7010879**		C-to-G < G-to-C NS
**SBS7b**	**As read on Coding or Non Transcribed Strand**							
		**To**						
	**From**	**A**	**T**	**C**	**G**	**Total**		A-to-T > T-to-A NS
	**A**		81	1066	0	**1147**	A<<T 1.4x p<0.001	A-to-G > T-to-C NS
	**T**	85		0	1565	**1650**		T-to-G >A-to-C 1.5x p<0.001
	**C**	642	9242		785	**10669**	G<<C 1.6x p<0.001	G-to-A < C-to-T 1.7x p<0.001
	**G**	5316	753	632		**6701**		G-to-T > C-to-A 1.2x p<0.05
						**20167**		C-to-G > G-to-C 1.2x p<0.01
**SBS7c**	**As read on Coding or Non Transcribed Strand**							
		**To**						
	**From**	**A**	**T**	**C**	**G**	**Total**		A-to-T < T-to-A 1.5x p<0.001
	**A**		63636	19723	14759	**98118**	A<<T 1.5x p<0.001	A-to-G < T-to-C 1.5x p<0.001
	**T**	94584		21529	27935	**144048**		T-to-G >A-to-C 1.4x p<p0.001
	**C**	3333	0		2758	**6091**	G<<C 1.4x p<0.001	G-to-A > C-to-T NS
	**G**	0	2489	1998		**4487**		G-to-T < C-to-A 1.3x p<0.001
						**252744**		C-to-G > G-to-C 1.4x p<0.001
**SBS7d**	**As read on Coding or Non Transcribed Strand**							
		**To**						
	**From**	**A**	**T**	**C**	**G**	**Total**		A-to-T < T-to-A NS
	**A**		146	2179	49636	**51961**	A<<T 1.6x p<0.001	A-to-G < T-to-C 1.6x p<0.001
	**T**	192		78908	2745	**81845**		T-to-G >A-to-C 1.3x p<0.001
	**C**	907	0		519	**1426**	G<<C 1.2x p<0.01	G-to-A > C-to-T NS
	**G**	0	1135	547		**1682**		G-to-T > C-to-A 1.3x p<0.001
						**136914**		C-to-G < G-to-C NS

Supplementary Table S4. Review of studies showing principal motif specificity of DNA and RNA oncogenic mutagens.

There are underlying caveats to the critical analysis reported here. Many cancer associated progression signatures (CPAS) are actually blunt 'deaminase overlay' composite signatures (Lindley et al 2016). Much of the COSMIC cancer mutation data analysed here has been sourced from advanced cancer patient samples. As such, it is likely that the statistical significance of the differences between mutation signatures analysed are blunted by the late-stage emergence of *de novo* cancer signatures predicting cancer progression (Lindley et al 2016, Mamrot et al 2019, Mamrot et al 2021). There is a need to be aware of these differences particularly in relation to animal cancer models which can be very misleading given the restricted APOBEC isoform repertoire in experimental animals compared to humans, with spontaneous canine cancer the closest real-world comparator for any pre-human clinical trials in immune oncology (Lindley and Steele 2019). Most deaminase signatures are tissue or tissue-group specific, while only some are ubiquitous (Lindley 2020). And the ADAR A-to-I RNA deaminases can also self-edit. This is reviewed in detail in Lindley (2020).

Mutagenic Agent	Motif	References
AID	WRC (GYW)	Beale et al (2004) Confirmed by many studies
APOBEC1<br>(RNA Editing – Lipid Metabolism; and DNA Deamination)	TCA (TGA)	Beale et al (2004) Confirmed by many studies<br>Potent DNA deaminase (mutator) in bacterial assay systems.
“APOBEC Signature Mutations in cancer”	TCW (WGA)	Roberts et al (2013) Often called “Kataegis” or a “storm” of clustered C- to-U deaminations
APOBEC3A<br>(Innate Immunity and RNA Editing)	YTCA (TGAR)	Chan et al (2015), Taylor et al (2013)<br>Logue et al (2014)
APOBEC3B<br>(Innate Immunity)	RTCA (TGAY)<br><br>RTCG (CGAY)	Roberts et al (2013), Chan et al (2015)<br>Taylor et al (2013), Leonard et al (2013), Burns et al (2013a)<br>Burns et al (2013b)
APOBEC3C<br>(Innate Immunity)	TC=CC > GC > AC<br>(GA = GG > GC > GT)	Yu et al (2004) - Retroviral restriction systems
APOBEC3D<br>(Innate Immunity)	TCG = TCT<br>= ACG = ACT<br>(CGA = AGA = CGT = AGT)	Dang et al (2006) - Retroviral restriction systems
APOBEC3F<br>(Innate Immunity)	TC (GA)	Yu et al (2004), Weigand et al (2004), Liddament et al (2004), Bishop et al (2004), Hache et al 2005), Miyagi et al. (2010) (Retroviral restriction)
APOBEC3G<br>(Innate Immunity)	CC (TC)<br>(GG, GA)	Beale et al (2004), Dang et al (2006), Henry et al (2009), Yu et al (2004), Wiegand et al (2004), Bishop et al (2004), Liddament et al (2004), Hache et al (2005) (Retroviral restriction)
APOBEC3H<br>(Innate Immunity)	TC (GA)	Dang et al (2006), Henry et al (2009), Chan et al (2015), Miyagi et al (2010)<br>Harari et al (2009) (Retroviral restriction)
ADAR1/2<br>(Signature enrichment)	WA (TW)	Bass (2002)

Footnote Table S4 : As indicated the many overlapping target motifs that reflect uncertainty in the field on the exact hierarchy of the targeting preferences of each listed motif. This summary table adapted from Table 1, Lindley (2020). In the motifs W = A/T, R = A/G and Y = C/T. Innate Immunity is indicated where retroviral and retroelement restriction has been demonstrated. APOBEC1 has demonstrative site-specific and promiscuous C-to-U RNA editing at 5' U<u>C</u> 3' and 5' A<u>C</u> 3' motifs (Sowden et al 1996; Blanc and Davidson 2003; Rosenberg et al 2011). APOBEC3A shown to have clear C-to-U editing in ssRNA (Cs in unpaired loops) substrates at 5' U<u>C</u> 3' motifs (Sharma et al 2015, 2016).

References

Alexandrov LB, Nik-Zainal S, Wedge DC, Aparicio SA, Behjati S, Biankin AV et al 2013 Signatures of mutational processes in human cancer Nature 2013 Vol 500 p415-421. DOI: 10.1038/nature12477

Alexandrov LB, Kim J, Haradhvala NJ,Huang MN, Ng AWT, Wu Yang, Boo A. 2020 The repertoire of mutational signatures in human cancer Nature 578: 94- 101, 2020

Anderson CJ, Talmane L, Luft J, Connelly J, Nicholson MD, Verburg JC, Pich O, et al 2024 Strand-resolved mutagenicity of DNA damage and repair Nature. 2024 Jun;630(8017):744-751.doi: 10.1038/s41586-024-07490-1. Epub 2024 Jun 12.

Aitken SJ, Anderson CJ, Connor F, Pich O, Sundaram V, Feig C, Rayner TF, et al 2020 Pervasive lesion segregation shapes cancer genome evolution. Nature. 2020 Jul;583(7815):265-270.doi: 10.1038/s41586-020-2435-1. Epub 2020 Jun 24.

Bass BL. (2002) RNA editing by adenosine deaminases that act on RNA Annu Rev Biochem. 2002:71:817-46. doi: 10.1146/annurev.biochem.71.110601.135501. Epub 2001 Nov 9.

Bayona-Feliu A, Herrera-Moyano E, Badra-Fajardo N, Galvan-Femenia I, Soler-Oliva ME, Aguilera A. 2023 The chromatin network helps prevent cancer-associated mutagenesis at transcription-replication conflicts. Nat Commun. 2023 Oct 28;14(1):6890.doi: 10.1038/s41467-023-42653-0.

Beale RCL, Petersen-Mahrt SK, Watt IN, Harris RS, Rada C, Neuberger MS. (2004) Comparison of the different context-dependence of DNA deamination by APOBEC enzymes: correlation with mutation spectra in vivo. J. Mol. Biol. 337 (2004) 585 - 596. doi: 10.1016/j.jmb.2004.01.046

Berry MW, Browne M, Langville AN, Pauca VP, and Plemmons RJ (2007). Algorithms and applications for approximate nonnegative matrix factorization. Comput. Stat. Data Anal. 52, 155–173.

Bishop KN, Holmes RK, Sheehy AM, Davidson NO, Cho SJ, Malim MH. (2004) Cytidine deamination of retroviral DNA by diverse APOBEC proteins. Curr. Biol. 14 (2004) 1392 – 1396. doi: 10.1016/j.cub.2004.06.057

Blanc V, Davidson NO. (2003) C-to-U RNA Editing: Mechanisms leading to genetic diversity. J. Biol. Chem. 278 (2003) 1395 – 1398. doi: 10.1074/jbc.R200024200

Buisson R, Langenbucher A, Bowen D, Kwan EE, Benes CH, Zou L, Lawrence MS. 2019 Passenger hotspot mutations in cancer driven by APOBEC3A and mesoscale genomic features. Science. 2019 Jun 28;364(6447):eaaw2872.doi: 10.1126/science.aaw2872.

Burns MB, Lackey L, Carpenter MA, Rathore A, Land AM, Leonard B, et al. (2013a) APOBEC3B is an enzymatic source of mutation in breast cancer. Nature 494 (2013) 366 - 371. doi: 10.1038/nature11881

Burns MB, Temiz NA, Harris RS. (2013b) Evidence for APOBEC3B mutagenesis in multiple human cancers. Nat Genet 45:977–983.

Chan K, Roberts SA, Klimczak LJ, Sterling JF, Saini N, Malc EP, et al. (2015) An APOBEC3A hypermutation signature is distinguishable from the signature of background mutagenesis by APOBEC3B in human cancers. Nat Genet. 2015 Sep;47(9):1067-72. doi: 10.1038/ng.3378.

Dang Y, Wang X, Esselman WJ, Zheng Y-H. (2006) Identification of APOBEC3-DE as another antiretroviral factor from the Human APOBEC Family J. Virol. 80 (2006) 10522 - 10533. doi: 10.1128/JVI.01123-06

Denissenko MF, Pao A, Tang M-s and Pfeifer GP (1996). Preferential formation of Benzo[a]pyrene adducts at lung cancer mutational hotspots in P53, Science 274, 430- 432.

Denissenko MF, Pao A, Pfeifer GP, Tang M-s. (1998). Slow repair of bulky DNA adducts along the nontranscribed strand of the human p53 gene may explain the strand bias of transversion mutations in cancers. Oncogene 16, 1241-1247.

Ewa B, Danuta M-S. 2017 Polycyclic aromatic hydrocarbons and PAH-related DNA adducts. J Appl Genet. 2017 Aug;58(3):321-330. doi: 10.1007/s13353-016-0380-3.Epub 2016 Dec 12.

Franklin A, Steele EJ. (2022) RNA-directed DNA repair and antibody somatic hypermutation. Trends in Genetics 38 (5): 426-436 doi: 10.1016/j.tig.2021.10.00. Epub 2021 Nov 2

Guttenplan JB, Kosinska W, Zhao Z-L, Chen K-M, Aliaga C, DelTondo J, et al 2012 Mutagenesis and carcinogenesis induced by dibenzo[a,l]pyrene in the mouse oral cavity: a potential new model for oral cancer. Int J Cancer. 2012 Jun 15;130(12):2783-90.doi: 10.1002/ijc.26344. Epub 2011 Nov 19.

Hache´ G, Liddament MT, Harris RS. (2005) The retroviral hypermutation specificity of APOBEC3F and APOBEC3G is governed by the C-terminal DNA Cytosine deaminase domain. J Biol. Chem 280 (2005) 10920 - 10924. doi: 10.1074/jbc.M500382200

Hanawalt PC, Spivak G. (2008). Transcriptional-coupled DNA repair: Two decades of progress and surprises, Nat. Rev. Mol. Cell. Biol. 9, 958-970.

Haradhvala NJ, Polak P, Stojanov P, Covington KR, Shinbrot E, Hess JM, et al. 2016 Mutational strand asymmetries in cancer genomes reveal mechanisms of DNA damage and repair. Cell. 2016;164(3): 538-549

Harari A, Ooms M, Mulder LC, Simon V. (2009) Polymorphisms and splice variants influence the antiretroviral activity of human APOBEC3H. J. Virol. 83 (2009) 295 - 3 03. doi: 10.1128/JVI.01665-08

Henry M, Guetard D, Suspene R, Rusniok C, Wain-Hobson S, et al. (2009) Genetic editing of HBV DNA by monodomain human APOBEC3 cytidine deaminases and the recombinant nature of APOBEC3G. PLoS ONE 4(1) (2009) e4277. doi :10.1371/journal.pone.0004277

Huang Y, Chen C, Russu IM. (2009) Dynamics and stability of individual base pairs in two homologous RNA-DNA hybrids. Biochemistry. 2009 May 12;48(18):3988-97.doi: 10.1021/bi900070f.

Ito F, Fu Y, Kao SA, Yang H, Chen XS. (2017)Family-Wide Comparative Analysis of Cytidine and Methylcytidine Deamination by Eleven Human APOBEC Proteins. J Mol Biol. 2017 Jun 16;429(12):1787-1799. doi: 10.1016/j.jmb.2017.04.021.

Kuraoka I, Endou M, Yamaguchi Y, Wada Y, Handa H, Tanaka K. (2003) Effects of endogenous DNA base lesions on transcription elongation by mammalian RNA polymerase II. J. Biol Chem 278,7294–72999. doi:10.1074/jbc. M208102200

Leonard B, Hart SN, Burns MB, Carpenter MA, Temiz NA, Rathore A, et al. (2013) APOBEC3B upregulation and genomic mutation patterns in serous ovarian carcinoma. Cancer Res. 73 (2013) 7222 - 7231. doi: 10.1158/0008-5472.CAN-13-1753

Liddament MT, Brown WL, Schumacher AJ, Harris RS. (2004) APOBEC3F properties and hypermutation preferences indicate activity against HIV-1 in vivo. Curr. Biol. 14 (2004) 1385 - 1391. doi: 10.1016/j.cub.2004.06.050

Lindley RA. (2013) The importance of codon context for understanding the Ig-like somatic hypermutation strand-biased patterns in TP53 mutations in breast cancer. Cancer Genet. 206 :222-226.

Lindley RA. (2020) Review of the mutational role of deaminases and the generation of a cognate molecular model to explain cancer mutation spectra. Med Res Arch. 8(8):2177. https://esmed.org/MRA/mra/article/view/2177

Lindley RA, Steele EJ. (2013) Critical analysis of strand-biased somatic mutation signatures in TP53 versus Ig genes, in genome -wide data and the etiology of cancer ISRN Genomics. Vol 2013 Article ID 921418, 18 pages.

Lindley RA, Steele EJ (2019) Deaminases and Why Mice Sometimes Lie in Immuno-Oncology Pre-Clinical Trials? Annals of Clinical Oncology. Hosting by Science Repository. http://dx.doi.org/10.31487/j.ACO.2019.01.001

Lindley RA, Steele EJ (2020) Presumptive Evidence for ADAR1 A-to-I Deamination at WA-sites as the Mutagenic Genomic Driver in Hepatocellular and Related ADAR1-Hi Cancers. J Carcinog Mutagen. 11:002. Available at : https://www.academia.edu/44713516/Presumptive_Evidence_for_ADAR1_A_to_I_Deamination_at_WA_sites_as_the_Mutagenic_Genomic_Driver_in_Hepatocellular_and_Related_ADAR1_Hi_Cancers

Lindley RA, Humbert P, Larner C, Akmeemana EH, Pendlebury CR. (2016) Association between targeted somatic mutation (TSM) signatures and HGS-OvCa progression. Cancer Med. 2016 Sep;5(9):2629-40. doi: 10.1002/cam4.825.

Logue EC, Bloch N, Dhuey E, Zhang R, Cao P, et al. (2014) A DNA sequence recognition loop on APOBEC3A controls substrate specificity. PLoS ONE 9(5) (2014) e97062. doi:10.1371/journal.pone.0097062

Luan DD, Korman MH, Jakubczak JL, Eichbush TH. (1993). Reverse transcription of R2B mRNA is primed by a nick at the chromosomal target site: a mechanism for non-LTR retrotransposition. Cell 72:595–605. doi:10.1016/0092-8674(93)90078-5

Malvezzi S, Farnung L, Aloisi CMN, Angelov T, Cramer P, Sturla SJ. 2017. Mechanism of RNA polymerase II stalling by DNA alkylation. Proc Natl Acad Sci USA. 2017 Nov 14;114(46):12172-12177.doi: 10.1073/pnas.1706592114.Epub 2017 Oct 30.

Mamrot J, Balachandran S, Steele EJ, Lindley RA. (2019) Molecular model linking Th2 polarized M2 tumour-associated macrophages with deaminase-mediated cancer progression mutation signatures. Scand J Immunol. 2019 May;89(5):e12760. doi: 10.1111/sji.12760.

Mamrot J, Hall NE, Lindley RA. (2021) Predicting clinical outcomes using cancer progression associated signatures. Oncotarget. 2021 Apr 13;12(8):845-858.doi: 10.18632/oncotarget.27934.

Mertz TM, Harcy V, Roberts SA. 2017 Risks at the DNA Replication Fork: Effects upon Carcinogenesis and Tumor Heterogeneity. Genes (Basel). 2017 Jan 22;8(1). pii: E46. doi: 10.3390/genes8010046.
Neil S, Bieniasz P (2009) Human Immunodeficiency Virus, Restriction Factors, and Interferon J. Interferon & Cytokine Research 29: 569-580

Otlu B, Diaz-Gay M, Vernes I, Bergstrom EN, Zhivagui M, Barnes M, Alexandrov LB. 2023 Topography of mutational signatures in human cancer. Cell Rep. 2023 Aug 29;42(8):112930.doi: 10.1016/j.celrep.2023.112930. Epub 2023 Aug 4.

Roberts SA, Lawrence MS, Klimczak LJ, Grimm SA, Fargo D, Stojanov P, et al. 2013 An APOBEC cytidine deaminase mutagenesis pattern is widespread in human cancers. Nat Genet 45:970–976.

Rosenberg BR, Hamilton CE, Mwangi MM, Dewell S, Papavasiliou FN. (2011) Transcriptome-wide sequencing reveals numerous APOBEC1 mRNA editing targets in transcript 3′ UTRs Nat Struct Mol Biol. 18 (2011) 230–236. doi:10.1038/nsmb.1975.

Sanchez A, Ortega P, Sakhtemani R, Manjunath L, Oh S, Bournique E, et al. 2024. Mesoscale DNA features impact APOBEC3A and APOBEC3B deaminase activity and shape tumor mutational landscapes. Nat Commun. 2024 Mar 18;15(1):2370.doi: 10.1038/s41467-024-45909-5.

Senigl F, Maman Y, Dinesh RK, Alinikula J, Seth RB, Pecnova L, et al. (2019). Topologically Associated Domains Delineate Susceptibility to Somatic Hypermutation . Cell Rep. 2019 Dec 17;29(12):3902-3915.e8. doi: 10.1016/j.celrep.2019.11.039.

Sharma S, Patnaik SK,Taggart RT, Kannisto ED, Enriquez SM, Gollnick P, Baysal BE. (2015) APOBEC3A cytidine deaminase induces RNA editing in monocytes and macrophages. Nat Commun 6 (2015) 6881 http://dx.doi.org/10.1038/ncomms7881

Sharma S, Patnaik SK, Kemera Z, Baysal BE. (2016) Transient overexpression of exogenous APOBEC3A causes C-to-U RNA editing of thousands of genes RNA Biology 14 (2016) 603 - 610. doi.org/10.1080/15476286.2016.1184387

Seplyarskiy VB, Soldatov RA, Popadin KY, Antonarakis SE, Bazykin GA, Nikolaev SI. 2016. APOBEC-induced mutations in human cancers are strongly enriched on the lagging DNA strand during replication. Genome Res. 2016 Feb;26(2):174-82.doi: 10.1101/gr.197046.115. Epub 2016 Jan 11.

Sowden M, Hamm JK, Smith HC. (1996) Overexpression of APOBEC-1 results in mooring sequence-dependent promiscuous RNA editing. J. Biol. Chem. 271 (1996) 3011 – 3017. http://www.jbc.org/content/271/6/3011.long

Steele EJ. (2009) Mechanism of somatic hypermutation: Critical analysis of strand biased mutation signatures at A:T and G:C base pairs. Molec. Immunol 46 : 305-320.

Steele EJ. (2016) Somatic hypermutation in immunity and cancer: Critical analysis of strand-biased and codon-context mutation signatures DNA Repair 45: 1-24.

Steele EJ, Franklin A, Lindley RA. 2024 Somatic mutation patterns at Ig and Non-Ig Loci. DNA Repair (Amst). 2024 Jan:133:103607.doi: 10.1016/j.dnarep.2023.103607.Epub 2023 Nov 28.

Steele EJ, Lindley RA. (2010) Somatic mutation patterns in non-lymphoid cancers resemble the strand biased somatic hypermutation spectra of antibody genes. DNA Repair. 9: 600-603

Swann PF. 1990 Why do O6-alkylguanine and O4-alkylthymine miscode? The relationship between the structure of DNA containing O6-alkylguanine and O4-alkylthymine and the mutagenic properties of these bases. Mutat Res. 1990 Nov-Dec;233(1-2):81-94.doi: 10.1016/0027-5107(90)90153-u.

Taylor BJM, Nik-Zainal S, Wu YL, Stebbings LA, Raine K, et al (2013) DNA deaminases induce break-associated mutation showers with implication of APOBEC3B and 3A in breast cancer kataegis. eLife 2 (2013) e00534. doi: 10.7554/eLife.00534

Thorslund T, Sunesen M, Bohr VA , Stevnsner T (2002) Repair of 8-oxoG is slower in endogenous nuclear genes than in mitochondrial DNA and is without strand bias. DNA Repair 1, pp. 261-273.

Wiegand HL, Doehle BP, Bogerd HP, Cullen BR. (2004) A second human antiretroviral factor, APOBEC3F, is suppressed by the HIV-1 and HIV-2 Vif proteins. EMBO J. 23 (2004) 2451 – 2458. doi: 10.1038/sj.emboj.7600246

Wirtz S, Nagel G, Eshkind L, Neurath MF, Samson LD, Kaina B. 2010. Both base excision repair and O6-methylguanine-DNA methyltransferase protect against methylation-induced colon carcinogenesis. Carcinogenesis. 2010 Dec;31(12):2111-7.doi: 10.1093/carcin/bgq174. Epub 2010 Aug 23.

Yu Q, Chen D, Konig R, Mariani R, Unutmaz D, Landau NR. (2004) APOBEC3B and APOBEC3C are potent inhibitors of simian immunodeficiency virus replication. J. Biol. Chem. 279 (2004) 53379 - 53386. doi: 10.1074/jbc.M408802200
